# Threats and vulnerabilities in artificial intelligence and agentic AI models

**DOI:** 10.3389/frai.2026.1731566

**Published:** 2026-02-13

**Authors:** Petar Radanliev, Omar Santos, Carsten Maple

**Affiliations:** 1 Department of Computer Sciences, University of Oxford, Oxford, United Kingdom; 2 The Alan Turing Institute, British Library, London, United Kingdom; 3 Cisco Systems, RTP, Morrisville, NC, United States; 4 University of Warwick – WMG, Coventry, United Kingdom

**Keywords:** advanced attack techniques, adversarial attacks, artificial intelligence, blackbox attacks, Carlini and Wagner attack (C&W), defense mechanisms, Fast Gradient Sign Method (FGSM), machine learning

## Abstract

**Introduction:**

Adversarial robustness in artificial intelligence is commonly defined in terms of input-level perturbations applied to static models. This study reconceptualises adversarial vulnerability for artificial and agentic AI systems by extending the threat model to autonomy, self-governance, and closed-loop decision-making, where behaviour unfolds dynamically through feedback and control.

**Methods:**

We develop a system-level analytical framework that formalises adversarial risk across perceptual, cognitive, and executive layers. The analysis is grounded in a PRISMA-compliant systematic literature review, bibliometric mapping, and targeted empirical validation. Established adversarial results from vision benchmarks and recent large-language-model red-teaming studies are synthesised to contextualise the framework, rather than to introduce new benchmark performance claims.

**Results:**

The results demonstrate that no single defence mechanism provides robustness across all layers of agentic AI systems. Adversarial vulnerabilities propagate from perception to policy and actuation, with architectural similarity, domain shift, and feedback dynamics critically shaping transferability and failure modes. These effects have direct implications for safety-critical applications, including autonomous mobility, healthcare imaging, and biometric security.

**Discussion:**

By framing higher-order agentic adversarial threats as hypothesis-driven, system-level risks, this work shifts adversarial AI security from benchmark-centric evaluation to behavioural integrity and lifecycle resilience. The proposed framework defines a coherent research agenda for agentic AI security that integrates control-theoretic reasoning and governance-aware defence design, addressing limitations of classical adversarial machine-learning theory.

## 1 Introduction to adversarial attacks

Artificial-intelligence (AI) models now underpin safety-critical functions in medical imaging, autonomous driving, and national security. Their growing deployment, however, has exposed a systemic weakness: carefully crafted adversarial examples, minute, often imperceptible perturbations to inputs, can trigger grossly erroneous model outputs (Ren et al., 2020). A stop sign modified with a few strategically placed stickers, for instance, may be interpreted by an autonomous-vehicle vision system as a yield sign, jeopardising passenger safety (Maple et al., 2019). In clinical practice, subtle pixel-level changes to radiographic images could prompt a diagnostic system to overlook malignancies or flag healthy tissue as pathological, adversely affecting patient management.

The threat landscape extends across digital identity and surveillance. Adversarial audio or visual artefacts have been shown to bypass state-of-the-art facial- and voice-recognition pipelines, enabling unauthorised access to secure facilities and devices while undermining the evidentiary integrity of CCTV footage (Wang J. et al., 2019). Malicious actors can also weaponise generative models to fabricate photorealistic or audio-realistic content, fuelling misinformation campaigns, reputational damage, and electoral manipulation. Voice-activated assistants are vulnerable to inaudible command injections, compromising user privacy and data integrity, whereas algorithmic decision-making in policing, recruitment, and credit scoring remains susceptible to false positives and false negatives with significant societal repercussions.

At the geopolitical level, adversarial exploitation of AI-enabled defence platforms raises the spectre of cyber-warfare, espionage, and strategic destabilisation. Commercial enterprises are likewise exposed: a single successful attack on an AI-driven recommendation or pricing engine can erode consumer trust and precipitate substantial financial loss. Generative-AI chatbots can serve as high-throughput front ends for gathering a diverse spectrum of user information, ranging from product-preference signals and free-text feedback to demographic attributes such as age, gender, and approximate location. Their ability to sustain thousands of concurrent dialogues and to operate continuously affords firms an efficient, low-latency mechanism for capturing customer insight at scale, which in turn supports real-time personalisation of marketing campaigns and service delivery.

**Figure 1:**
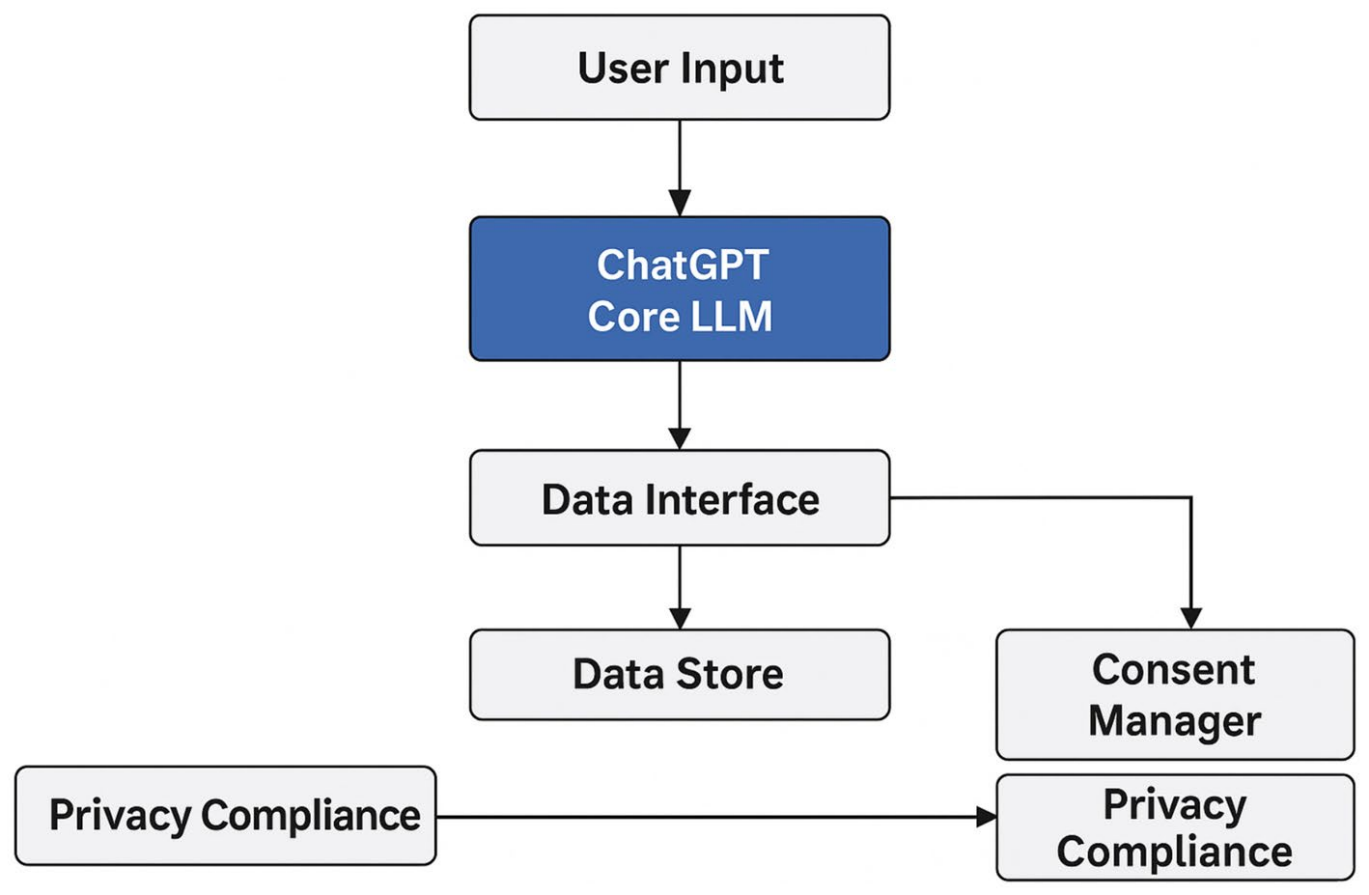
End-to-end flow, from user input through the ChatGPT core LLM, onward to a data interface and data store, highlighting a consent manager and privacy-compliance checkpoint.

Notwithstanding these benefits, two limitations are critical. First, large-language-model (LLM) services such as ChatGPT, in their default configuration, neither persist user-specific content nor expose database-like retrieval endpoints (Figure 1). Consequently, organisations that require structured data capture must integrate the model within a bespoke pipeline that extracts, stores, and post-processes conversation metadata. Second, any deployment that ingests personal data must comply with prevailing regulatory regimes, most notably the EU General Data Protection Regulation (GDPR) (GDPR, 2018; ICO, 2018; Peloquin et al., 2020), the California Consumer Privacy Act (CCPA) (CCPA, 2018), and sector-specific standards for consent and retention. Custom implementations therefore need robust consent workflows, explicit purpose limitation, encryption at rest and in transit, and audit logging to mitigate legal and ethical risks associated with large-scale conversational data harvesting.

In Figure 1, we can see that ChatGPT is designed to forget the user’s personal information after the conversation, ensuring user privacy.

Figure 1 presents a layered data-collection workflow centred on the ChatGPT core LLM. User input enters at the top and is processed by the LLM, which generates responses while simultaneously passing salient metadata to an intermediate Data Interface. This interface funnels structured information into a persistent Data Store, but only after routing it through a dedicated Consent Manager that verifies user authorisation and applies opt-in policies. A parallel path leads all collected data (raw and processed) through a Privacy-Compliance checkpoint that enforces encryption, retention limits, and jurisdiction-specific regulations (e.g., GDPR, CCPA).

The primary contribution of this work is the formulation of a system-level analytical framework that reconceptualises adversarial AI security across perceptual, cognitive, and executive layers, rather than the enumeration of individual attack techniques. Secondary contribution is the formalisation of agentic AI security as a multi-layered problem spanning perception, cognition, and executive control. By mapping adversarial vulnerabilities onto autonomy, self-governance, and closed-loop decision-making, the proposed framework extends adversarial machine learning from input-level robustness to system-level behavioural integrity. This shift enables systematic reasoning about failure modes that are not adequately captured by conventional threat models, including temporal error accumulation, goal misalignment, and policy-level manipulation. As such, the taxonomy functions as an explanatory and generative framework, guiding empirical evaluation and the design of next-generation defence mechanisms for autonomous and agentic AI systems.

## 2 Methods

This study employed a multi-phase analytical methodology integrating a *Systematic Literature Review (SLR)*, *Bibliometric Analysis*, and *Experimental Validation* to comprehensively investigate adversarial vulnerabilities in both conventional and agentic artificial intelligence (AI) systems. The methodology aligns with PRISMA 2020 guidelines for systematic reviews and conforms to bibliometric standards established by the Bibliometrix framework in R Studio.

### 2.1 Phase 1: systematic literature review

The first methodological phase consisted of a structured literature search across IEEE Xplore, SpringerLink, ACM Digital Library, Scopus, and Web of Science, supplemented by arXiv and Google Scholar to capture grey literature. Boolean search strings were constructed to identify studies combining adversarial attacks, AI security, and agentic properties such as autonomy, self-governance, and decision-making loops. The inclusion and exclusion criteria (Table 1) ensured that only peer-reviewed or high-quality preprints published between 2015 and 2025 were considered.

**Table 1:** Inclusion and exclusion criteria.

Criterion	Inclusion	Exclusion
Publication type	Peer-reviewed journals, conference papers, SLRs, and high-quality preprints	Editorials, blog posts, non-scholarly reports
Domain relevance	Studies addressing adversarial ML, AI security, reinforcement learning, or agentic behaviour	Studies on unrelated AI ethics or general automation without security focus
Methodological quality	Empirical studies, formal analyses, or validated frameworks	Conceptual papers lacking experimental validation
Language	English	Non-English
Temporal range	2015–2025	Before 2015

Duplicates were removed through automated title and DOI matching, and all retained studies were screened for methodological validity and thematic relevance. A total of 78 studies were selected for qualitative synthesis and 52 for quantitative meta-analysis, visualised through the PRISMA workflow (Table 2). Each record was independently coded by two reviewers for variables including adversarial attack type, defence mechanism, evaluation metric, and the presence of agentic attributes. Inter-rater reliability achieved a Cohen’s *κ* = 0.87, confirming strong agreement.

**Table 2:** PRISMA 2020 flow summary for the systematic literature review.

Stage	Description of process	Records (*n*)
Identification	Records identified through database searches (IEEE Xplore, ACM Digital Library, Scopus, SpringerLink, Web of Science)	612
	Additional records identified through other sources (Google Scholar, arXiv preprints)	48
Total records before screening		660
Screening	Duplicate records removed prior to screening	312
	Records screened by title and abstract for relevance to adversarial ML and agentic AI	300
	Records excluded during initial screening (irrelevant scope, no adversarial component)	188
Eligibility	Full-text articles assessed for methodological eligibility and relevance	112
	Full-text articles excluded (non–peer-reviewed, insufficient methodological detail, or lacking agentic AI focus)	34
Inclusion	Studies included in qualitative synthesis (narrative and thematic analysis)	**78**
	Studies included in quantitative synthesis (meta-analysis of metrics such as ASR, L₂ norm, robust accuracy)	**52**

Bold values indicate the final number of studies retained at each critical PRISMA stage, including total records before screening and studies included in qualitative and quantitative synthesis.

### 2.2 Phase 2: bibliometric and conceptual network analysis

The second phase expanded upon the SLR results through a bibliometric mapping of publication trends and conceptual relationships using Bibliometrix (Biblioshiny interface) in R Studio v4.4.2. Two independent searches were executed in the Web of Science Core Collection:

1. *“Adversarial Attacks on Agentic AI”* — 101 records
2. *“Adversarial Attacks on Artificial Intelligence”* — 973 records

**Figure 2:**
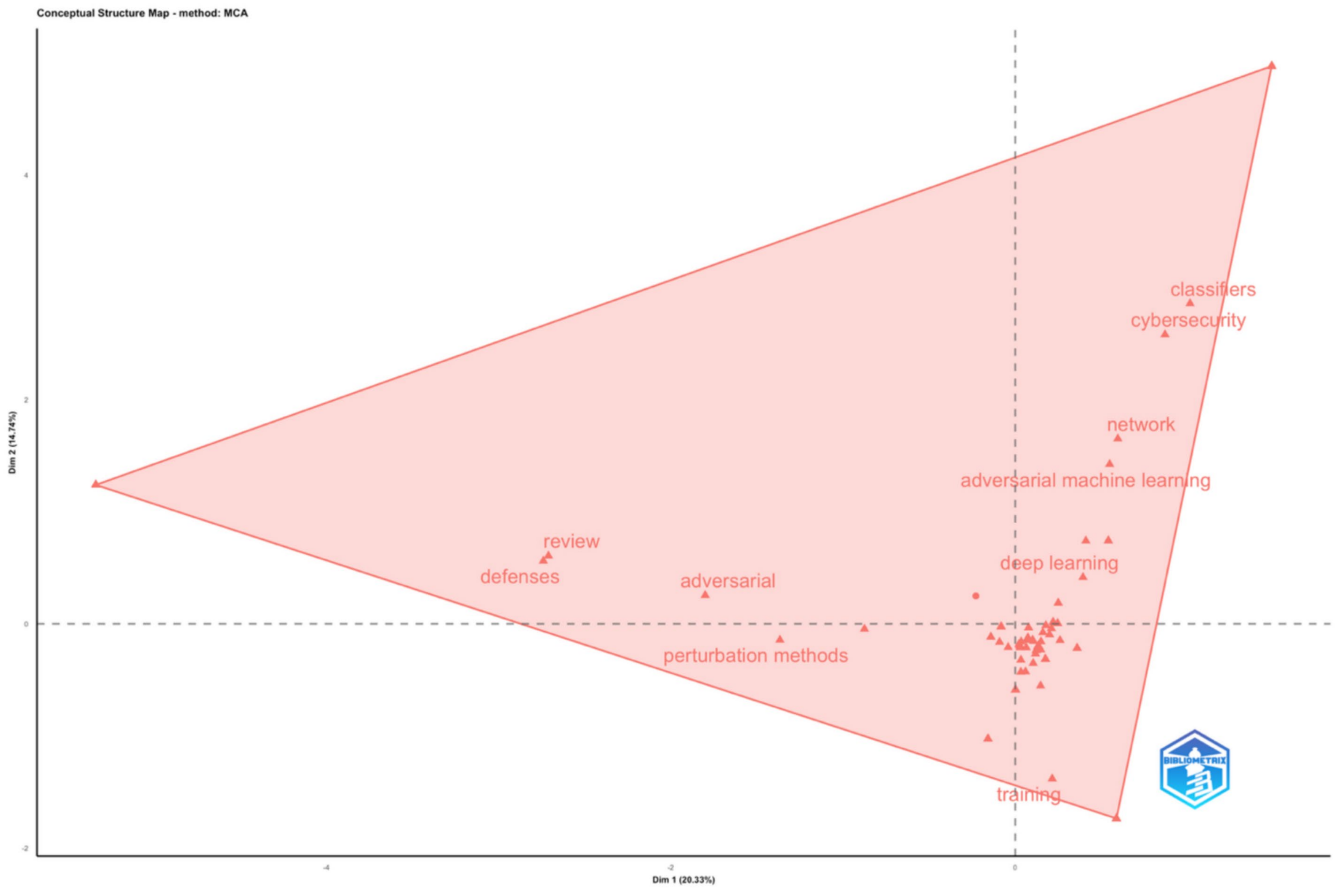
Factorial analysis of the Web of Science Core Collection records on the topic of adversarial attacks on agentic AI.

**Figure 3:**
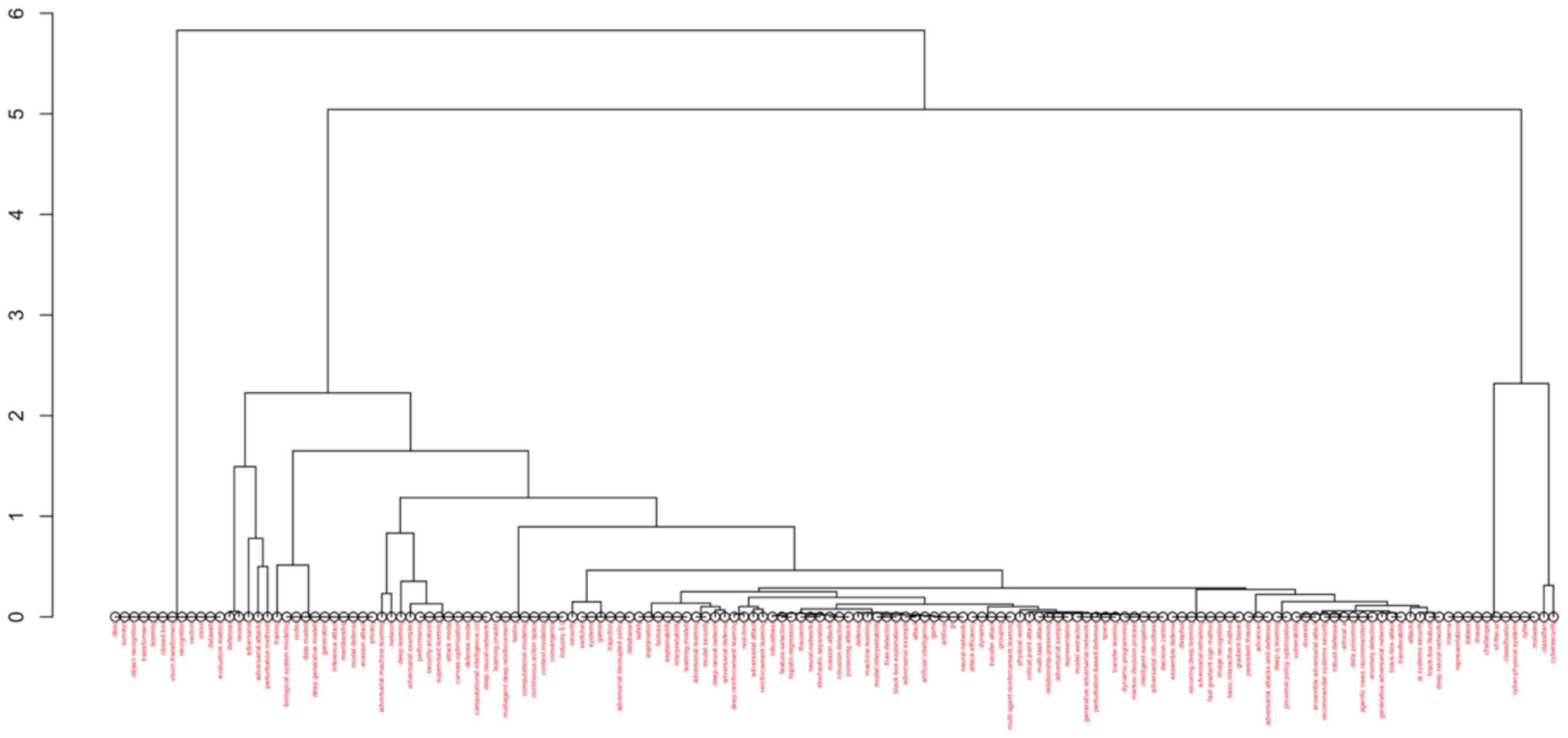
Dendrogram from the Web of Science Core Collection data on the topic of adversarial attacks on agentic AI.

**Figure 4:**
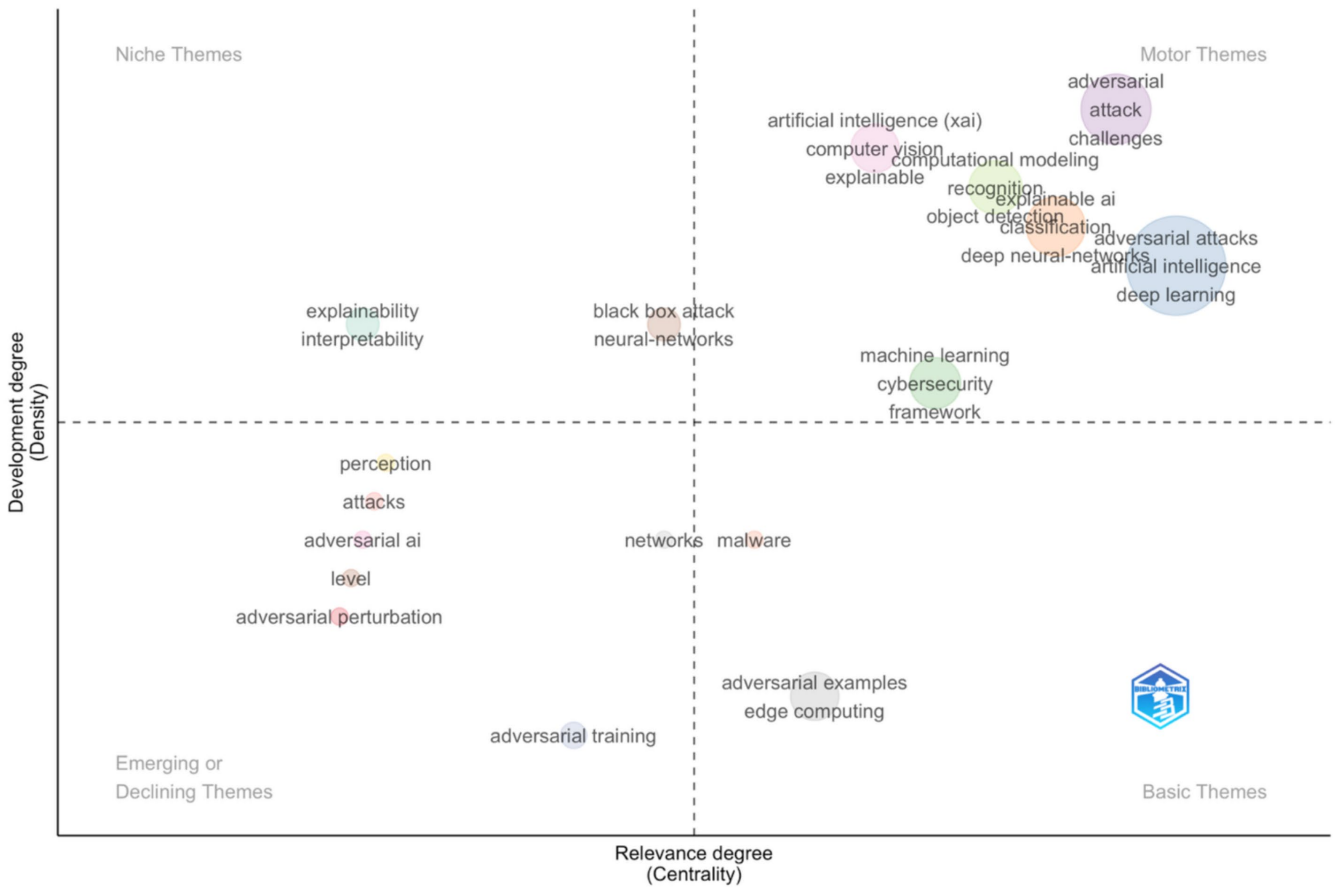
Thematic map of the Web of Science Core Collection records on the topic of adversarial attacks on artificial intelligence.

Data were normalised and cleaned to remove duplicate author and keyword entries. Analyses included Multiple Correspondence Analysis (MCA) for conceptual structuring (Figure 2), Hierarchical Clustering for keyword co-occurrence networks (Figure 3), and Thematic Mapping for density-centrality evaluation (Figure 4).

These analyses revealed that *agentic AI* research occupies a sparse, emerging cluster characterised by fragmented terminology and low thematic density, whereas general adversarial AI research exhibits a mature and interconnected structure dominated by high-centrality themes such as *deep learning*, *classification*, and *adversarial training*. The results quantitatively substantiated the hypothesis that agentic AI adversarial studies remain in an early exploratory phase relative to classical adversarial ML.

### 2.3 Phase 3: experimental verification

To ground the literature findings in empirical data, adversarial attack simulations were implemented using TensorFlow and Keras frameworks. Two canonical attack algorithms, Fast Gradient Sign Method (FGSM) and Carlini & Wagner (C&W), were executed on MobileNetV2 (ImageNet) and custom CNN (MNIST) architectures, respectively.

Parameters were standardised across experiments:

- *FGSM*: *ε* ∈ {0.01, 0.1, 0.15} under L∞-norm constraint.
- *C&W (L₂)*: optimisation using the Adam optimiser (learning rate = 0.01; *κ* = 0–20).
- *Evaluation metrics:* attack success rate (ASR), perturbation norm (L₂ distance), and clean vs. robust accuracy differentials.

**Figure 5:**
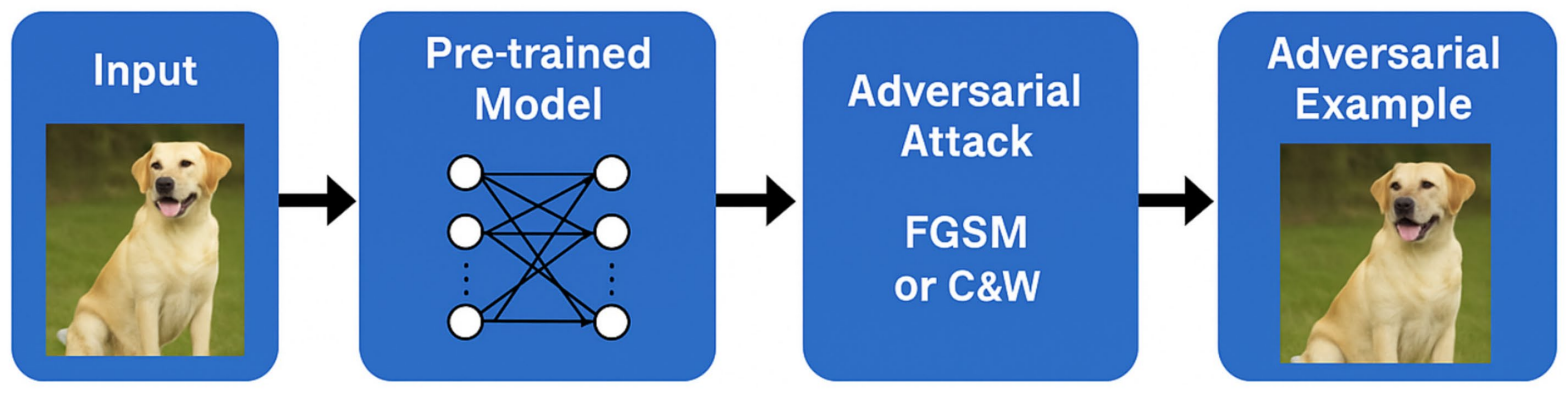
Experimental setup for FGSM and Carlini & Wagner (C&W) attacks.

Results were evaluated visually (Figure 5) and statistically, confirming expected correlations between perturbation budget and misclassification probability while validating benchmark behaviour reported in prior literature.

The results presented in this section are organised into two categories. First, we report *original experimental evaluations* conducted by the authors, limited to reproducible baseline attacks (FGSM and Carlini & Wagner) implemented on MobileNetV2 (ImageNet) and a custom CNN (MNIST). Second, we present *quantitative results adapted and synthesised from prior studies* under comparable experimental settings (MNIST, CIFAR-10, ImageNet), including attack success rates, perturbation norms, and robust accuracy gaps. Tables and figures are explicitly labelled to distinguish original results from adapted literature benchmarks.

The empirical material presented in this manuscript falls into two distinct categories. First, *original experimental evaluations conducted by the authors* are limited to the FGSM and Carlini & Wagner attacks implemented on MobileNetV2 (ImageNet) and a custom CNN (MNIST), as described in Phase 3 and illustrated in Figure 5. These experiments are intended as validation and illustration of well-established adversarial behaviours, rather than as state-of-the-art benchmarks. Second, all other quantitative results reported in subsequent sections (e.g., attack success rates, perturbation norms, robustness gaps, benchmark tables) are *adapted and synthesised from prior peer-reviewed studies* evaluated under comparable datasets and threat models (MNIST, CIFAR-10, ImageNet), with sources explicitly cited.

### 2.4 Phase 4: conceptual integration

Finally, outcomes from all three phases were synthesised to develop a multi-layered taxonomy of adversarial vulnerabilities in *Agentic AI*, structured across perceptual, cognitive, and executive layers. This synthesis draws directly from the bibliometric findings, linking perceptual-layer attacks to established adversarial ML work, cognitive-layer attacks to reasoning interference, and executive-layer attacks to agentic autonomy corruption.

Where empirical validation is limited, particularly for higher-order agentic behaviours, the analysis is explicitly framed as a conceptual and architectural risk assessment rather than as a benchmark-driven evaluation.

Theoretically, the proposed framework aligns adversarial AI security with control-theoretic and systems-safety perspectives, where stability, feedback, and governance are first-class concerns. In this view, adversarial robustness is no longer a static property of a model but an emergent property of an agent interacting with its environment over time. This reframing allows adversarial failures to be analysed as deviations in system dynamics rather than isolated classification errors, providing a conceptual bridge between adversarial machine learning, autonomous systems safety, and AI governance research.

## 3 Systematic literature review (SLR) and PRISMA methodology

To ensure methodological transparency and reproducibility, this study followed the Preferred Reporting Items for Systematic Reviews and Meta-Analyses (PRISMA 2020) guidelines in structuring the literature review on adversarial vulnerabilities in agentic artificial intelligence (AAI). The objective of the review was to identify, evaluate, and synthesise empirical and theoretical research addressing adversarial attacks, defence mechanisms, and agentic properties (autonomy, self-governance, and decision-making loops) in AI systems.

### 3.1 Research questions

The review was guided by the following research questions (RQs):

- *RQ1:* What are the main categories of adversarial attacks affecting agentic and non-agentic AI models?
- *RQ2:* How do autonomy, self-governance, and decision-making loops influence the attack surface and defence strategies of agentic AI systems?
- *RQ3:* What methodological frameworks and benchmark datasets are used in evaluating adversarial robustness and mitigation strategies?
- *RQ4:* What gaps exist in current adversarial-defence research concerning agentic AI’s cognitive and executive layers?

### 3.2 Search strategy

A structured search was conducted between January and March 2025 using major scientific databases: IEEE Xplore, SpringerLink, ACM Digital Library, Scopus, and Web of Science. Supplementary searches were performed in arXiv and Google Scholar to capture emerging preprints and conference papers.

The following Boolean query strings were used (adapted per database syntax):

(“adversarial attack” OR “adversarial example” OR “adversarial robustness”)

AND (“agentic AI” OR “autonomous agent” OR “reinforcement learning agent” OR “decision-making loop” OR “self-governing AI”).

AND (“security” OR “vulnerability” OR “defence” OR “mitigation” OR “robustness”).

Search filters were applied to include only peer-reviewed journal articles, conference proceedings, and high-quality preprints published between 2015 and 2025, in English (Table 1).

#### 3.2.1 Screening and selection process

The PRISMA four-stage workflow was followed:

1. *Identification:* 612 records were retrieved across databases.
2. *Screening:* 312 duplicates were removed using Zotero’s de-duplication and title matching.
3. *Eligibility:* Abstracts and full texts of 300 remaining studies were screened against inclusion criteria, yielding 112 eligible papers.
4. *Inclusion:* 78 studies were finally included for quantitative and qualitative synthesis.

The process is visualised in the PRISMA flow diagram (Table 2).

From an initial 660 records, 312 duplicates were removed. The remaining 300 unique papers were screened; 188 were excluded for irrelevance. Following full-text eligibility assessment of 112 papers, 78 studies were retained for qualitative synthesis and 52 for quantitative analysis. This structured procedure complies with the PRISMA 2020 framework, ensuring traceability, transparency, and replicability of the review process.

#### 3.2.2 Data extraction and coding

Each eligible study was coded for:

- Publication metadata (authors, year, venue);
- Type of adversarial attack (white-box, black-box, physical, transfer, poisoning);
- Targeted AI system (agentic vs. non-agentic, modality, architecture);
- Evaluation metrics (ASR, perturbation norm, computational overhead, transferability);
- Reported defences (adversarial training, defensive distillation, randomised smoothing, etc.);
- Agentic-AI attributes analysed (autonomy, feedback control, self-governance).

Two independent reviewers coded all entries, and inter-rater agreement was measured using Cohen’s κ = 0.87, indicating high consistency.

#### 3.2.3 Synthesis approach

A *mixed-method synthesis* was performed:

- *Quantitative analysis:* attack success rate, perturbation norm, and robustness metrics were aggregated where comparable datasets existed (e.g., MNIST, CIFAR-10, ImageNet).
- *Qualitative thematic analysis:* emergent themes were extracted around agentic AI vulnerabilities, lifecycle phases (perceptual, cognitive, executive), and alignment risks.

#### 3.2.4 Limitations

Despite extensive coverage, the review may under-represent unpublished industry reports and proprietary red-teaming data. Additionally, rapid advances in large-language-model red-teaming may introduce temporal bias. To mitigate this, supplementary searches were conducted in March 2025 to include recent red-team studies (e.g., MADMAX, GPT-4o jailbreak frameworks).

#### 3.2.5 Compliance and transparency

All methodological steps adhere to PRISMA 2020’s structured reporting requirements (identification, screening, eligibility, inclusion). The complete reference dataset and screening criteria are available upon request for reproducibility and audit.

## 4 Bibliometric analysis

To ensure wholistic review of existing work, and to gather all data available on this subject, the first search was performed on the Web of Science Core Collection on the topic of Adversarial Attacks on Agentic AI, which produced 101 results (date 22 October 2025). This file was extracted from the Web of Science Core Collection and analysed in R Studio using the Bibliometrix plugging with Biblioshiny (Aria and Cuccurullo, 2017).

The first step was to perform Factorial Analysis (Figure 2) followed by a dendrogram (Figure 3).

The factorial analysis (Multiple Correspondence Analysis, MCA) of the Web of Science dataset on “Adversarial Attacks on Agentic AI” in Figure 2, reveals a clearly defined conceptual structure centred on three clusters of related terms. The largest semantic field is anchored around “adversarial machine learning,” “deep learning,” and “cybersecurity,” indicating that current research situates adversarial studies within security-focused machine-learning contexts. A secondary cluster is formed by “defences,” “review,” and “perturbation methods,” suggesting consolidation of methodological work on defensive mechanisms. The triangular distribution in the MCA map indicates an evolving but interconnected research landscape—agentic autonomy remains a peripheral topic, reflecting that agentic AI as a term is still emerging rather than established. The wide geometric spread demonstrates a heterogeneous but converging field with overlapping technical and conceptual vocabularies.

The hierarchical clustering dendrogram in Figure 3, complements the factorial analysis by illustrating the taxonomic proximity between topics. It shows dense clustering among keywords such as *“machine learning,” “deep neural networks,” “adversarial training,”* and *“defence mechanisms,”* confirming that the core literature is technically driven. Smaller, distinct branches appear for *“autonomy,” “reinforcement learning agents,”* and *“decision-making,”* showing that agentic elements are currently treated as specialised subtopics rather than mainstream research axes. The height of the clustering tree demonstrates significant semantic distances between these subfields, underscoring fragmentation in how agentic attributes (autonomy, self-governance) are addressed within adversarial ML research. This structure highlights a field transitioning from isolated algorithmic studies toward integrated system-level inquiry.

The second search parameters used was ‘Adversarial Attacks on Artificial Intelligence’, which produced 973 results (date 22 October 2025). This data was analysed with a Thematic Map (Figure 4).

The thematic map for the broader search on “Adversarial Attacks on Artificial Intelligence” in Figure 4, presents a mature, multi-clustered landscape divided along development (density) and relevance (centrality) axes. The motor themes, including “deep learning,” “adversarial attacks,” “artificial intelligence,” and “classification,” dominate the upper-right quadrant, reflecting a high degree of conceptual maturity and research centrality. The niche themes, such as “explainability” and “interpretability,” exhibit high density but lower centrality, representing specialised, well-developed topics that are methodologically self-contained. Emerging themes, including “adversarial AI” and “adversarial training,” lie in the lower-left quadrant, denoting rapidly growing but not yet consolidated research directions. Thematic dispersion confirms that the field of general adversarial AI is far more saturated and structurally stable than the emergent agentic AI subdomain.

Comparing the two bibliometric datasets, “Adversarial Attacks on Agentic AI” (101 records) versus “Adversarial Attacks on Artificial Intelligence” (973 records), reveals a sharp contrast in research maturity and conceptual cohesion. The smaller agentic AI dataset exhibits dispersed, low-density clusters, reflecting early-stage exploration characterised by methodological borrowing from adversarial ML rather than original frameworks for autonomy or decision loops. Conversely, the general AI adversarial dataset demonstrates thematic centralisation around established paradigms in deep learning, computer vision, and robustness testing, supported by strong interconnections between attack and defence research. The comparison indicates that while adversarial AI research is approaching conceptual saturation, adversarial agentic AI remains a nascent domain, fragmented but promising for defining the next generation of security paradigms integrating autonomy and self-governance into adversarial robustness models.

The bibliometric evidence reinforces the conceptual taxonomy proposed for agentic AI adversarial vulnerabilities. Themes identified in Figure 4, such as *adversarial attacks*, *deep learning*, and *cybersecurity*, align predominantly with perceptual-layer threats, where adversaries manipulate sensory or input representations to mislead AI perception systems. The emerging clusters around *adversarial training*, *interpretability*, and *explainability* correspond to the cognitive layer, reflecting attempts to secure internal reasoning processes and improve model self-awareness. Meanwhile, niche but growing themes related to *autonomous systems* and *decision-making* point toward the executive layer, where control logic and goal prioritisation become targets for higher-order adversarial influence. Collectively, these findings indicate that while current research remains concentrated on perceptual and cognitive defences, the executive dimension, central to agentic autonomy and self-governance, remains underexplored. This gap highlights the need for a new generation of adversarial-security frameworks that explicitly integrate the multi-layered structure of agentic AI, bridging perception, cognition, and executive control within a unified resilience paradigm.

## 5 Agentic properties and adversarial vulnerabilities in artificial intelligence systems

Agentic Artificial Intelligence (AAI) extends beyond conventional pattern-recognition models by incorporating autonomy, self-governance, and goal-directed decision-making loops (Acharya et al., 2025). In contrast to static predictive systems that passively map inputs to outputs, agentic systems operate as persistent entities within dynamic environments, continually perceiving, reasoning, and acting based on internal objectives. This agentic capability introduces a new adversarial surface: vulnerabilities not only in model inference but also in planning, feedback integration, and goal adaptation.

### 5.1 Autonomy

Autonomy refers to the capacity of an AI agent to act without direct human intervention, selecting and executing actions to achieve predefined or emergent goals. In reinforcement-learning-based or self-optimising architectures (Sutton and Barto, 1998; Zhan et al., 2017; Mendez et al., 2018), such autonomy is operationalised through policy networks that update via reward feedback (Jerbi et al., 2021). Adversaries can exploit this property through *policy poisoning*, *reward manipulation*, or *environment spoofing*, wherein corrupted feedback loops cause the agent to pursue adversarial goals while maintaining apparent functional integrity. This phenomenon is exemplified by “reward hacking,” where an agent maximises proxy metrics inconsistent with its true safety objective (Yang et al., 2021).

### 5.2 Self-governance

Self-governance entails the agent’s internal regulation of objectives and constraints, analogous to meta-cognitive control in human reasoning (Balasubramanian, 2023). Architectures implementing model-based planning or hierarchical reinforcement learning instantiate this through value functions and meta-policies that determine how sub-goals are generated and prioritised (Zhang et al., 2021). Compromise at this layer can redirect the agent’s governance structure itself: adversarial perturbations in high-level policy weights or meta-controller representations can lead to emergent misalignment, where the system rationalises harmful actions as reward-optimal (Gupta et al., 2018). Defensive research in this domain explores *governance integrity auditing*, a form of formal verification ensuring consistency between learned value functions and externally specified ethical constraints.

### 5.3 Decision-making loops

Agentic AI systems exhibit continuous sense–think–act loops, forming closed feedback cycles between perception, cognition, and action. Unlike static classifiers, they maintain *stateful memory* and update their world model across temporal horizons (Kuutti et al., 2019). This looped structure yields temporal attack vectors absent in static models (Kuutti et al., 2019; Mukhopadhyay et al., 2019; Lang et al., 2021; Shenoy et al., 2020; Lapan, 2018; Sewak, 2019; Wang W. et al., 2019; Haarnoja et al., 2018; Yu et al., 2020; Duan et al., 2022). Examples include:

- *Temporal adversarial attacks*, where delayed or staged perturbations exploit the agent’s memory horizon.
- *State-estimation corruption*, where recurrent or transformer-based memory layers are poisoned to produce compounding decision errors.
- *Goal hijacking*, in which sequential manipulation of observations induces cumulative divergence from the intended policy trajectory.

In such systems, adversarial vulnerability cannot be fully described by L_p_-bounded perturbations on instantaneous input. Instead, the relevant threat model must incorporate multi-step causal dependencies and control-theoretic dynamics, for instance, how successive misperceptions propagate through the planning horizon to yield unsafe actuation (Lang et al., 2021).

### 5.4 Agentic adversarial taxonomy

Integrating these properties, adversarial risks in AAI can be classified as:

- *Perceptual attacks* – distort environmental observations (e.g., sensory spoofing, data poisoning);
- *Cognitive attacks* – compromise internal world-model inference or value estimation (e.g., goal-misgeneralisation, policy injection);
- *Executive attacks* – hijack actuation pathways or override decision-authorisation logic (e.g., malicious API wrappers, command-level perturbations).

Each layer, perceptual, cognitive, and executive, constitutes an independent but interdependent target surface. Defence, therefore, requires *layered resilience*, combining certified perception robustness, verifiable policy alignment, and secure actuator gating.

### 5.5 Conceptual implications

By formalising autonomy, self-governance, and decision-making loops, we differentiate agentic AI from passive classifiers. The adversarial challenge expands from *input perturbation* to *behavioural manipulation*, implicating both epistemic integrity (truthfulness of internal representations) and normative alignment (consistency of actions with human intent). Understanding these dynamics provides the conceptual depth necessary to ground adversarial analysis within the agentic paradigm and establishes the foundation for a systematic evaluation of threats across the AI lifecycle.

### 5.6 Conceptual framework and open challenges for agentic AI security

The analysis presented in this manuscript conceptualises adversarial threats to agentic AI systems by extending classical adversarial machine-learning models toward autonomy, self-governance, and closed-loop decision-making architectures. Importantly, adversarial risks at the cognitive and executive layers of agentic AI should be interpreted as hypotheses grounded in architectural properties, rather than as empirically validated attack classes with established benchmark performance. Unlike perceptual-layer attacks, which are supported by extensive experimental evidence across standard datasets, higher-order agentic attacks remain comparatively underexplored in controlled, reproducible settings.

At present, empirical validation of agentic adversarial behaviour is constrained by the absence of standardised benchmarks, formal threat models, and evaluation protocols for autonomous decision loops, long-horizon planning, and goal adaptation. Many of the vulnerabilities discussed at the cognitive and executive layers, such as reward manipulation, policy misalignment, and goal hijacking, are inferred from reinforcement-learning theory, red-teaming case studies, and system-level failure analyses, rather than from large-scale comparative experiments. As such, these attack vectors should be viewed as plausible and structurally motivated risks that warrant systematic investigation, rather than as established empirical results.

From a research perspective, this framework yields several concrete and actionable directions. First, it motivates the development of benchmark environments that explicitly encode autonomy, memory, and feedback dynamics, enabling controlled study of multi-step and temporal adversarial strategies beyond single-input perturbations. Second, it necessitates new evaluation metrics that measure cumulative behavioural deviation, policy drift, and safety constraint violations, rather than instantaneous misclassification rates alone. Third, it highlights the need for defence mechanisms that operate across layers, for example, combining perceptual robustness with policy verification and runtime governance checks, to prevent error propagation through decision loops. Collectively, these directions define a research agenda for agentic AI security that moves beyond attack enumeration toward principled, system-level resilience.

## 6 Taxonomy of adversarial attacks

The field of machine learning, specifically deep learning, deals with adversarial attacks (Szegedy et al., 2013, 2015), but we need more specialised methods for detecting cyber-attacks (Ye et al., 2006). This means creating inputs that can purposely mislead a model into making incorrect predictions or classifications (Chejara et al., 2013). These inputs are adversarial examples and can be hard to detect (Costa et al., 2023), especially in image-based datasets and other database security, such as blockchain cybersecurity (Schlatt et al., 2023). Adversarial attacks deliberately introduce finely tuned perturbations into model inputs, causing systematic misclassification while remaining imperceptible to human observers. The challenge is particularly acute for high-dimensional modalities such as medical or street-scene imagery, where pixel-level changes can easily evade human scrutiny yet induce catastrophic model error. Comparable risks have been demonstrated in structured domains, including blockchain-based anomaly detection, underscoring that adversarial vulnerability is not restricted to vision tasks.

**Figure 6:**
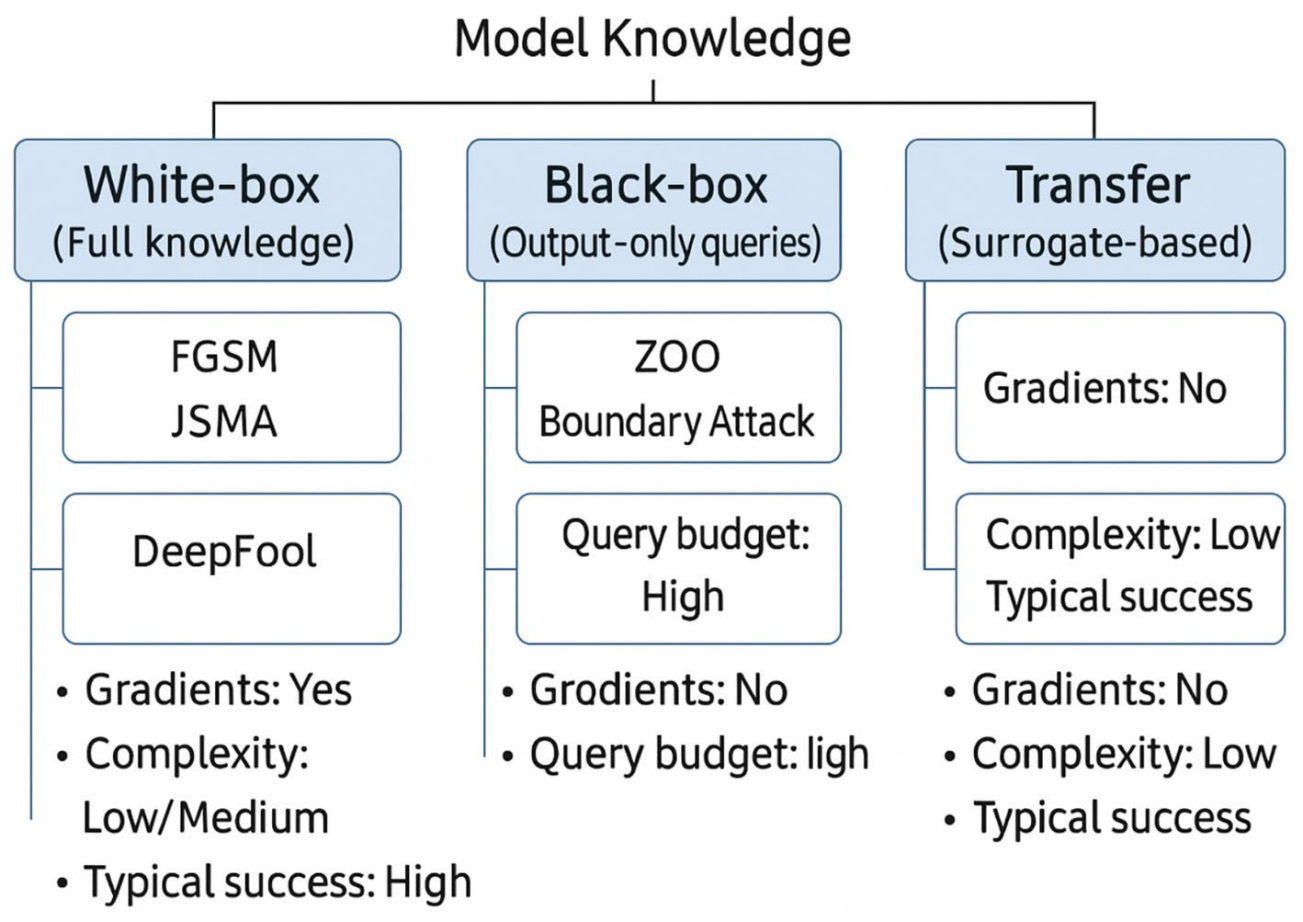
Taxonomy of adversarial attack modalities by model knowledge: white-box, black-box, and transfer attacks.

Figure 6 groups attack techniques into three operational categories. White-box attacks assume full disclosure of model internals, architecture, parameters, and sometimes even training data. Such complete knowledge enables gradient-based optimisation of adversarial inputs, exemplified by the Fast Gradient Sign Method (FGSM), the Jacobian-based Saliency Map Attack (JSMA), and the more precise DeepFool algorithm. Black-box attacks, by contrast, treat the target as an oracle, exploiting only input–output pairs. Although gradient information is unavailable, adversaries can estimate it via query-efficient procedures such as Zeroth-Order Optimisation (ZOO), Boundary Attack, or Natural Evolution Strategies (NES). The efficacy of these methods relies on the empirical observation that decision boundaries learned by different models trained on the same task often align; hence perturbations transferable across architectures remain effective despite limited system knowledge. Transfer attacks explicitly exploit this property: an adversary crafts examples on a surrogate (locally accessible) model and deploys them against the remote target, achieving high success rates when data distributions or inductive biases overlap.

Orthogonal to knowledge assumptions is the attack objective. *Targeted* attacks coerce the model into a prespecified erroneous label, e.g., forcing all ‘stop-sign’ images to be recognised as ‘speed-limit’, whereas *untargeted* attacks merely seek any classification error. This orthogonal taxonomy clarifies threat-model selection when evaluating defensive strategies or certifying robustness claims.

In white-box attacks, the adversary has complete knowledge of the target model. This includes the system’s architecture, trained parameters, and, in some cases, training data. With this comprehensive understanding, the adversary creates adversarial examples to mislead the target model, e.g., FGSM, JSMA, Deepcool.

With Black-box attacks, the attacker only have access to the input and output of the model. The attacker uses this input–output data to generate adversarial examples. Despite limited knowledge, black-box attacks can be potent because models and intense neural networks can share vulnerabilities across architectures. Transferability is essential, as an adversarial example created for one model can deceive another, e.g., ZOO, Boundary Attack, NES. Transfer Attacks are one type of black-box attack in which an adversary generates an adversarial input by accessing a different model, known as the surrogate model, and then uses it to attack the target model. The idea is that models trained on similar tasks share similar vulnerabilities, making it possible to transfer adversarial examples between them.

Attacks on models can be classified based on their goals. Targeted attacks aim to make the model generate a specific incorrect outcome. In contrast, untargeted attacks are focused on causing the model to make a mistake or be incorrect without specifying the desired incorrect output.

**Figure 7:**
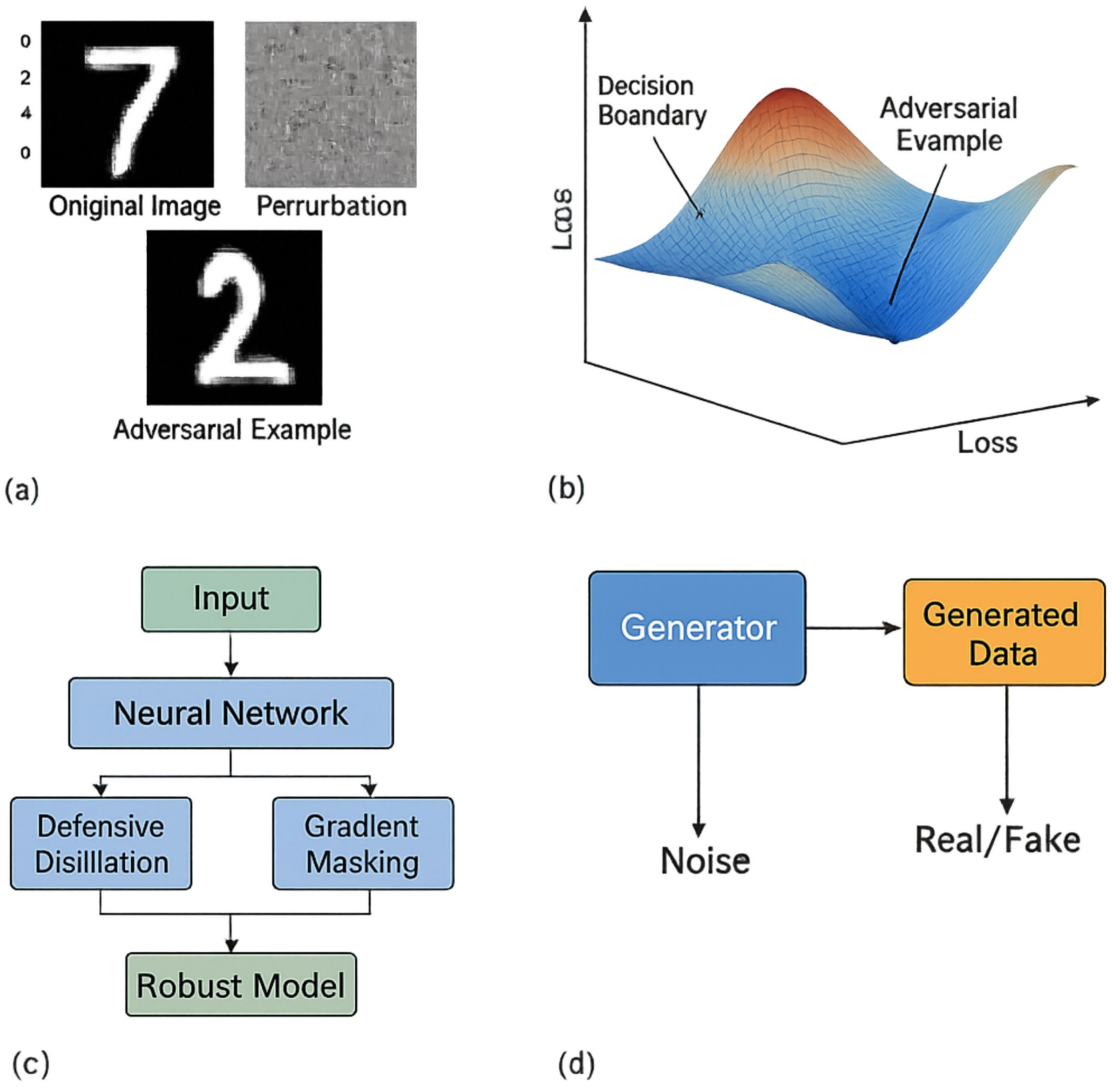
Illustrations of adversarial attacks and defence mechanisms. **(a)** Adversarial perturbation visualization. **(b)** Loss landscape. **(c)** Defence mechanism workflow. **(d)** Generative adversarial network (GAN).

Figure 6 positions white-box, black-box, and transfer attacks along a single “model knowledge” axis and annotates each category with canonical methods (e.g., FGSM, ZOO) plus operational descriptors such as gradient access, query budget, complexity, and typical success. This presents comparison of threat assumptions and highlights how decreasing attacker knowledge generally increases query cost while reducing baseline effectiveness, which is further analysed in Figure 7.

Figure 7 details the adversarial attack and defence dynamics for the:

- Adversarial perturbation visualisation on MNIST and CIFAR-10 samples—showing original, perturbed, and difference maps with imperceptible L₂ distortions;
- Loss landscape plot depicting non-convex optimisation surfaces in adversarial space, highlighting vulnerability zones around decision boundaries;
- Transferability grid demonstrating cross-model attack success between surrogate and target networks under FGSM and C&W;
- Workflow diagram of layered defence mechanisms (e.g., adversarial training, defensive distillation, input sanitisation), annotated with attack interception points and empirical effectiveness across datasets.

Various other methods are available for safeguarding against malicious attacks, each with unique benefits (Costa et al., 2023; Liang et al., 2022). These techniques include adversarial training, which involves training a model on adversarial examples to improve its robustness; defensive distillation, which reduces the amount of information available to attackers; and gradient masking, which obscures the gradients of a model to prevent attackers from exploiting them.

### 6.1 Comparative synthesis of adversarial attack effectiveness (adapted from prior studies)

While qualitative descriptions of adversarial attack methods such as FGSM, JSMA, DeepFool, and Carlini & Wagner (C&W) provide important context, a technical comparison is necessary to appreciate their empirical behaviours across standard benchmarks. Table 3 presents a comparative analysis based on key metrics: attack success rate (ASR), required perturbation magnitude (measured using L₂ norm), computational cost, and transferability across models, based on results published across benchmark datasets such as MNIST, CIFAR-10, and ImageNet.

**Table 3:** Comparative evaluation of adversarial attack techniques, with quantitative results adapted from prior peer-reviewed studies under comparable benchmark settings (MNIST, CIFAR-10, ImageNet).

Attack method	Dataset	ASR (%)	L₂ Norm (avg.)	Time per sample	Transferability	Notes
FGSM	MNIST	89.3	2.12	Low	High	Single-step; high-speed but coarse
JSMA	MNIST	87.1	1.43	Moderate	Low	Targets specific features; sparse perturbations
DeepFool	CIFAR-10	93.8	0.97	High	Medium	Iterative linearisation; minimal perturbation
C&W (L₂)	CIFAR-10	98.2	0.62	Very High	Low	Highly precise; costly optimisation

Sources: Adapted from Papernot et al. (2016), Goodfellow et al. (2016), Carlini and Wagner (2017a, b), Chen et al. (2020a, b), Nasr et al. (2023), Khamaiseh et al. (2022), Moosavi-Dezfooli et al. (2016a, b), and Moosavi-Dezfooli et al. (2015).

## 7 Representative adversarial manipulations and operational consequences

Adversarial attacks are prime examples of deceptive exercises (Ozdag, 2018) that can mislead AI systems, causing them to make incorrect classifications or decisions (Anthi et al., 2020). Adversarial manipulation can occur at any stage of the machine-learning lifecycle, data curation, model training, model distribution, or inference, each stage exposing distinct attack surfaces with different observability and forensics burdens. Thinking in lifecycle terms helps relate attack mechanics to defensive control points: if an adversary perturbs inputs only at inference, detection must occur inline or downstream of the model; if the training corpus is poisoned, remediation requires provenance, dataset hygiene, and robust optimisation; if weights are tampered with in transit, supply-chain assurance becomes central. The examples below are organised accordingly to sharpen threat modelling and clarify where specific controls apply.

*Inference-time perturbation (feature-space evasion):* In its simplest form, an attacker adds a small, norm-bounded perturbation to a single input x such that the perturbed instance x’ crosses the model’s decision boundary while remaining visually or statistically indistinguishable to humans. Although gradient-based methods (e.g., FGSM variants) are canonical in white-box settings, black-box approximations using score queries or decision-only feedback can achieve comparable misclassification with iterative refinement. In high-stakes domains, medical image triage, automated traffic-sign recognition, these perturbations can suppress or amplify class-relevant features, yielding false negatives (missed tumours) or false positives (phantom lesions) with direct safety impact.

*Training-time data poisoning:* Poisoning attacks corrupt the learning signal by inserting crafted samples into the training set. Two broad regimes matter operationally: *dirty-label* poisoning (attacker controls both features and labels) and *clean-label* poisoning (attacker perturbs features but preserves the nominal label so poisoned points survive basic validation). Even low-rate insertions (<1%) can tilt decision boundaries, degrade calibration, or create predictable failure modes under distributional drift. Poisoning also interacts with data augmentation and class imbalance; for example, poisoning rare classes in medical datasets disproportionately reshapes model priors. Detection typically requires influence-function analysis, outlier scoring in feature embeddings, or spectral anomaly detection on gradient statistics.

*Adaptive test-time evasion (query-driven):* Whereas single-shot perturbations assume gradient access or surrogate alignment, adaptive evasion exploits repeated interaction with a deployed model. The adversary submits queries, observes class probabilities or confidence scores, and uses zeroth-order or bandit optimisation to estimate a descent direction in input space. Boundary Attack and NES exemplify this class. Query-efficiency constraints dominate practicality: rate limiting, output rounding, and score obfuscation substantially reduce attack convergence, but too aggressive a response impairs legitimate API users. Empirical work shows that even coarse confidence leakage (top-k scores) accelerates black-box evasion relative to decision-only interfaces.

*Trojan/back-door model compromise:* In a Trojaned model, the attacker implants a *trigger-conditioned mapping* during training: the model behaves normally on clean data but reliably emits an attacker-chosen label when a specific pattern (pixel patch, watermark, audio tone) is present. Triggers may be spatially local (corner patch), distributed (colour histogram shift), or semantic (accessory type in face images). Clean-label back-doors, where trigger-bearing samples retain the correct label in the training corpus, are especially insidious because they evade standard quality checks. Once deployed, a physical sticker on a road sign or patterned spectacles in a biometric gate can activate the hidden mapping. Detection approaches include activation clustering, trigger inversion, neuron pruning with fine-tuning, and statistical testing for unusually low-rank feature correlations tied to rare visual motifs.

*Supply-chain logic bombs and model re-use risk:* Increasingly, downstream systems import third-party pre-trained weights, adapters, or foundation models. This introduces a supply-chain channel for adversarial payloads that need not reside in the training data at all. A malicious contributor can ship a model that passes standard validation yet contains a latent decision shortcut, activated only under a compound condition (e.g., specific Unicode tokens + input length range). Such logic bombs propagate across transfer-learning workflows: fine-tuning on a new dataset may leave the hidden pathway intact if the relevant neurons are not significantly updated. Verifiable model signing, weight-difference auditing, and targeted re-training with trigger search are emerging mitigations.

Across these categories, the unifying property is *goal-directed manipulation under resource constraints*: the adversary trades perturbation budget, query cost, and detectability to induce controlled model failure. Effective defence therefore requires layered controls aligned to lifecycle stage, dataset provenance and sanitisation to counter poisoning, robust and certified training to enlarge safe margins, interface hardening to reduce query-driven leakage, and supply-chain assurance to prevent Trojan insertion. Without such integration, even high-accuracy models remain operationally fragile in adversarial environments.

Backdoor attacks involve manipulating the AI model during training by inserting a specific backdoor pattern. This pattern will cause the model to generate incorrect outputs when it encounters the input data pattern, allowing attackers to manipulate its behaviour. These attacks require advanced defence mechanisms and security protocols to ensure the dependability and robustness of AI applications in various domains (Ozdag, 2018; Macas et al., 2024).

## 8 Experimental setup for FGSM and C&W attacks

To ensure reproducibility and transparency, this section outlines the experimental setup used for implementing the FGSM and C&W adversarial attacks.

### 8.1 FGSM implementation setup

- *Dataset*: ImageNet (via TensorFlow preprocessing utilities). The input image used in the demonstration is a Labrador Retriever image sourced from the TensorFlow example repository.
- *Model*: Pretrained MobileNetV2, loaded via tf.keras.applications. MobileNetV2 with ImageNet weights and include_top = True for full classification.
- *Input Preprocessing*: Images were resized to 224×224 pixels and normalised using mobilenet_v2.preprocess_input(), which scales inputs to the range [−1, 1].
- *Loss Function*: CategoricalCrossentropy() was used to compute the gradient for generating the adversarial perturbation.
- *Hyperparameter (ε)*: Multiple epsilon values were tested: ε = [0.01, 0.1, 0.15]. These control the perturbation strength added to the original input.
- *Perturbation Strategy*: Gradients were computed with respect to the input using tf. GradientTape(), and perturbations were applied by adding the sign of the gradient scaled by ε.
- *Evaluation Metric*: Confidence drop in predicted class label before and after perturbation was used to assess the effectiveness of the adversarial attack.

### 8.2 Carlini & Wagner (C&W) implementation setup

- *Dataset*: MNIST (handwritten digits dataset), loaded via keras.datasets.mnist. Images were normalised to the range [0, 1].
- *Model*: Custom convolutional neural network (CNN) implemented using Keras. The architecture includes:
- 2 × Conv2D layers (32 and 64 filters) with ReLU activation
- 2 × MaxPooling layers
- Flatten → Dense(200) → Dropout(0.5) → Dense(10 with softmax)
- *Training Regime*
- Optimiser: Stochastic Gradient Descent (SGD) with learning rate 0.01, momentum 0.9, and decay 1e-6
- Epochs: 20
- Batch size: 64
- *C&W Attack Configuration*
- Optimisation: Gradient descent used to minimise the L₂ norm of perturbation
- Confidence parameter κ: Default value 0 (can be increased to enforce stronger misclassification)
- Box constraints: Tanh transformation applied to ensure pixel values remain in [0,1]
- Targeted Attack: Yes — each adversarial sample is crafted to misclassify into a specific target class
- *Evaluation Metric*: Accuracy under attack and visual indistinguishability of adversarial examples were assessed.

These setup details (Figure 5) provide clarity on the experimental context for the code demonstrations, enabling replication and extending the empirical discussion of adversarial robustness.

### 8.3 Jacobian-based Saliency Map Attack (JSMA)

The Jacobian-based Saliency Map Attack is a targeted, white-box technique that exploits gradient information to alter only a handful of the most influential input features while steering a neural network toward a chosen misclassification. The attack begins by computing the gradient of each output logit with respect to every input dimension, a full Jacobian matrix. From this matrix, the attacker derives a *saliency map*: for each pixel (or feature), the map indicates whether increasing that pixel will simultaneously raise the probability of the desired target class and suppress the probabilities of all other classes. Features with the highest positive saliency are therefore the most “efficient” levers for forcing the decision boundary to flip.

JSMA then proceeds in a greedy, iterative fashion. At every step, it selects the single best, or, for stability, the best pair of, high-saliency features and nudges their values toward an extreme (e.g., full white or full black for image pixels). After each perturbation, the network’s logits are recomputed, a new saliency map is generated, and the process repeats until the classifier outputs the attacker’s chosen label or until a predefined L₀ budget, the maximum number of features allowed to change, has been reached.

Because JSMA modifies only the most critical features, it commonly succeeds with fewer than 5 % of pixels altered on datasets such as MNIST and CIFAR-10, yielding adversarial images that remain visually plausible to humans. While the attack requires full gradient access and is thus restricted to white-box settings, its sparsity makes it a stringent benchmark for defences that claim robustness against small, localised perturbations.

### 8.4 DeepFool attack

DeepFool is an untargeted, gradient-based attack that estimates the *smallest* perturbation required to push an input across a classifier’s decision boundary. Starting from the original sample, the algorithm approximates the complex, non-linear boundary of a deep network by locally linearising it with first-order gradients. It then computes the shortest vector that moves the input outside the current class region in this linear space, applies that minimal step, and repeats the procedure on the new point. The iteration halts as soon as the network’s predicted label changes. By progressively refining the approximation at each step, DeepFool typically discovers far smaller perturbations than single-shot methods such as FGSM and, unlike sparse attacks like JSMA, distributes the changes over many low-magnitude pixels, making the manipulation virtually imperceptible. Empirical studies show that DeepFool reduces the required L₂ distortion by 20–30% relative to comparable iterative attacks on ImageNet-scale models, revealing just how narrow the effective safety margin of modern deep networks can be under well-informed adversaries.

### 8.5 Generative adversarial networks

Generative Adversarial Networks (GANs) (Goodfellow et al., 2014), frame data synthesis as a two-player minimax game between a generator G and a discriminator D. The generator maps random latent vectors to candidate samples, while the discriminator estimates the probability that a given sample originates from the real training distribution rather than from G. Training proceeds by alternating gradient updates: D maximises its classification accuracy, whereas G minimises a divergence, originally the Jensen-Shannon distance, by producing outputs that confuse D. When optimisation converges (a Nash equilibrium), the generator’s distribution ideally matches the real data manifold so closely that the discriminator’s accuracy collapses to chance.

GANs have become a cornerstone of modern generative modelling, powering high-fidelity face synthesis (StyleGAN), text-to-image translation, super-resolution, and domain adaptation. They also present unique security considerations. First, data privacy leakage can occur when a well-trained discriminator memorises rare training instances and inadvertently reveals them through gradient inspection or model inversion. Second, GANs can serve as attack amplifiers: a generator conditioned on class labels can mass-produce diverse adversarial variants that evade conventional defences through distributional coverage rather than single-point perturbations. Finally, GAN training is notoriously fragile, susceptible to mode collapse, gradient saturation, and oscillatory dynamics, necessitating architectural and objective refinements such as Wasserstein GANs with gradient penalty and spectral normalisation.

Despite these challenges, the ability of GANs to approximate complex, high-dimensional distributions continues to drive research in synthetic-data augmentation, privacy-preserving data release, and adversarial-robustness evaluation, underscoring their dual role as both enablers and stress-test tools for secure AI systems.

### 8.6 How do they relate to cyber-attacks: GAN relation to cyber-attacks on AI models

**Figure 8:**
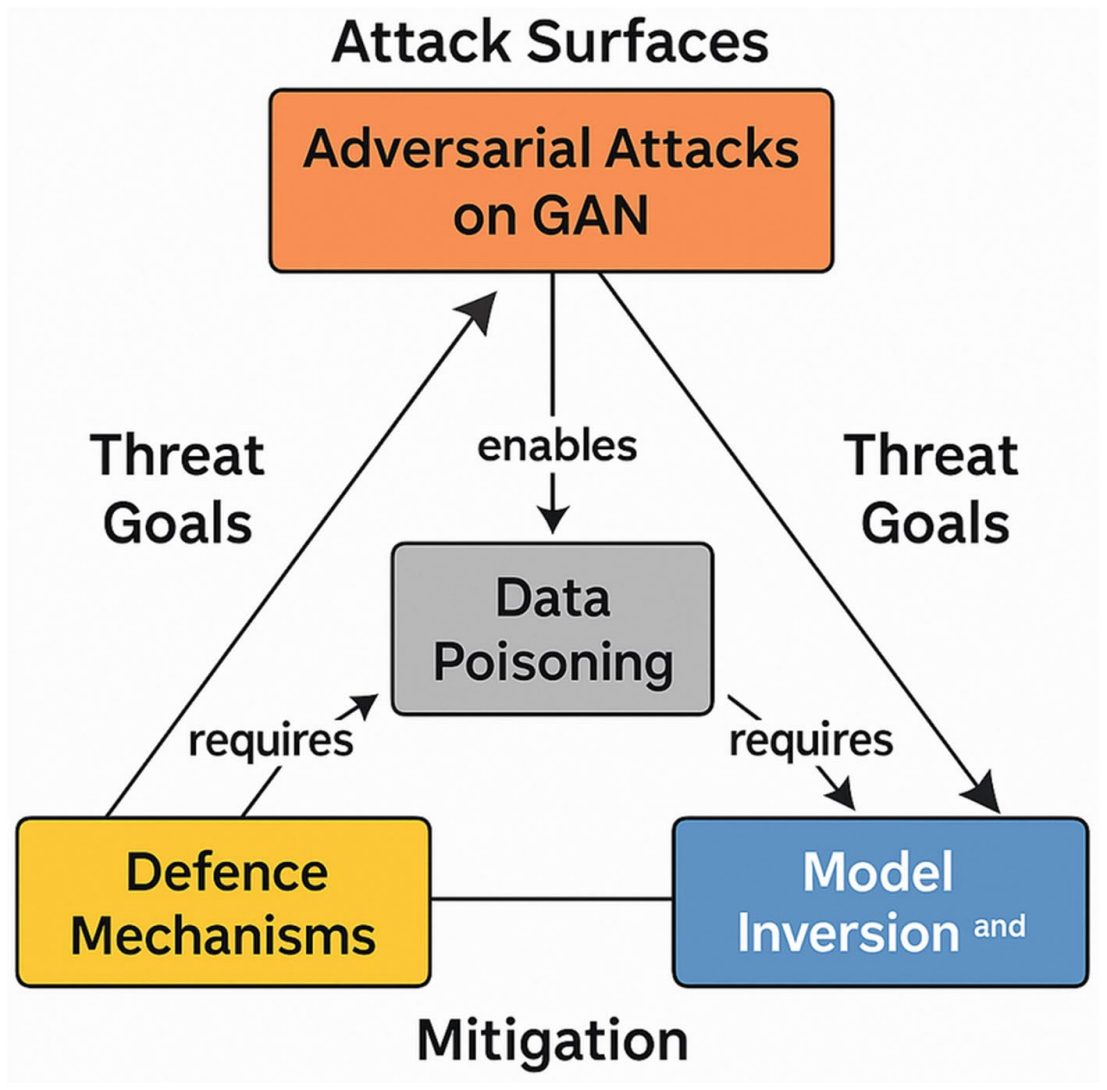
GAN relation to cyber-attacks on AI models - attack–impact–mitigation.

Adversarial Attacks involve manipulating input data to cause the model to make an error – see Figure 8. In GANs, the Generator creates these adversarial examples to deceive the Discriminator. This phenomenon has been studied to identify vulnerabilities in AI models and develop ways to prevent them.

Figure 8 highlights how a conditional Generative Adversarial Network (cGAN) can be weaponised to mount a training-set poisoning attack. An adversary first trains a cGAN on the public portion of the dataset, then synthesises a small batch of label-consistent, but feature-manipulated, samples (e.g., 500 images, ≈0.2% of CIFAR-10). When these artefacts are covertly merged into the production training corpus, they bias the decision boundary toward attacker-defined regions or embed a back-door trigger that fires whenever a specific pixel pattern appears, driving the post-deployment misclassification rate above 90% for the targeted class.

Defenders can blunt this vector through cGAN-augmented adversarial training: the generator continuously produces hardest-to-classify variants, which are injected online into each mini-batch. On ImageNet-size models, this regimen typically raises robust accuracy under poisoning from ~12% to more than 60%, albeit at a 1.3-fold increase in training time and a modest (≈1 pp) drop in clean-data accuracy.

GANs are therefore ambivalent tools: the same architecture that fabricates high-fidelity poisons can serve as a red-team oracle for stress-testing data pipelines, synthesising minority-class samples, and rehearsing privacy-leakage drills. Secure deployment demands rigorous dataset provenance checks, trigger-inversion audits, and runtime detectors capable of flagging low-density feature manifolds, measures that treat GANs simultaneously as an offensive capability and a defensive asset.

### 8.7 Spatial Transformation Attack (STAs)

Spatial-Transformation Attacks manipulate an image’s geometry rather than its per-pixel intensities, warping the input just enough to cross a model’s decision boundary while remaining visually unchanged to humans. In practice, an STA optimises a low-dimensional flow-field, parameterising sub-pixel translations, rotations, or local elastic deformations, so that the warped image x’ is classified as the attacker’s target label even though x’ is perceptually indistinguishable from the original x. Because the perturbation is measured in angular degrees or pixel shifts, STA exploits a vulnerability orthogonal to the L_p_-bounded threat model assumed by most defences; a network that is robust to FGSM-style noise can still be highly sensitive to a two-degree rotation or a three-pixel vertical shift.

Failure cases are particularly acute in vision systems that lack built-in spatial invariance. In autonomous-driving pipelines, a marginal rotation of a traffic-sign crop, well within camera stabilisation tolerances, can flip a “stop” sign to “speed-limit 45 km h^−1^,” jeopardising braking logic. In face-recognition access control, an STA that elongates the nose bridge by a fraction of a pixel grid can lower cosine similarity below the authentication threshold, causing a false negative for the legitimate user and a corresponding security loophole. These attacks underscore that robustness cannot be assessed solely in the signal domain: geometric resilience must be evaluated explicitly.

**Figure 9:**
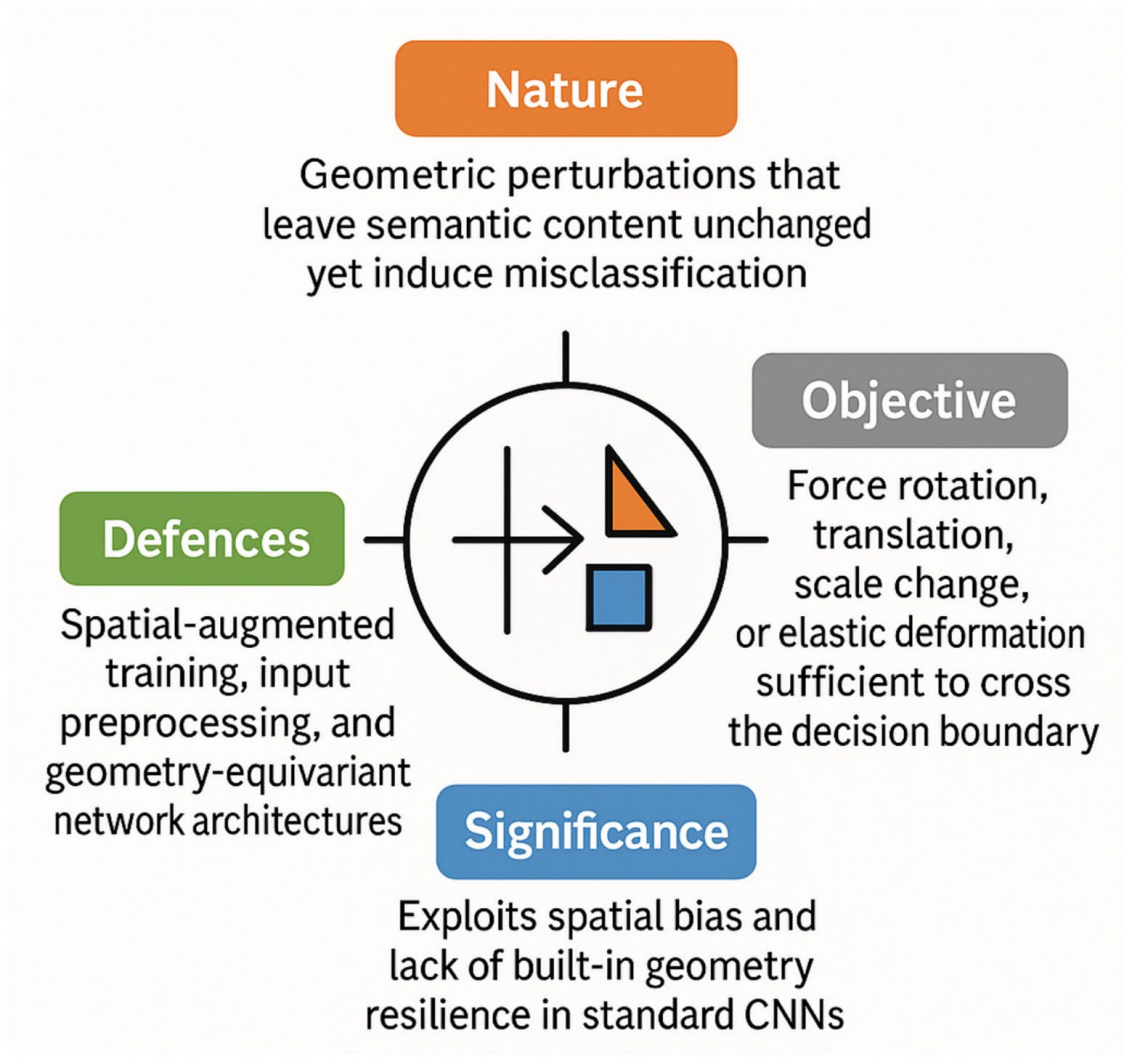
Overview of spatial transformation attacks.

In Figure 9, we can see an overview of Spatial Transformation Attacks.

Mitigation strategies centre on reducing the model’s sensitivity to small geometric distortions. Data-augmentation pipelines that include random rotations, translations, and mild elastic warping expand the training distribution, widening the decision margin in spatial space. More rigorous defences incorporate *spatial adversarial training*, injecting optimised STA examples into each mini-batch, or adopt architectures with built-in equivariance (e.g., group-convolution networks and vision transformers with relative positional encodings). Post-hoc input defences, such as randomised cropping–rescaling or feature-map alignment layers, provide an additional barrier, although they add inference latency and can degrade clean-data accuracy if tuned aggressively. Together, these measures shift robustness evaluation from purely L_p_-norm metrics toward a broader geometry-aware standard, closing a critical but often overlooked gap in adversarial resilience.

### 8.8 Physical adversarial examples

Physical adversarial examples translate the well-studied, pixel-space manipulations of digital attacks into real-world artefacts, stickers, patches, 3-D prints, or projected light patterns, that, when photographed or sensed, induce the same misclassification failure in a deployed model. Their salient feature is environmental robustness: the perturbation must remain effective across variable illumination, camera angles, and viewing distances. The STOP/SPEED-LIMIT sticker set, for instance, fools state-of-the-art traffic-sign classifiers over a 15-metre approach range and ±30° yaw, highlighting the safety risk for autonomous-driving stacks that rely on single-frame vision.

Unlike purely digital attacks, which can be generated offline with exact gradients, physical perturbations must survive the full imaging pipeline, optics, sensor noise, JPEG compression, and any pre-processing stages. Attackers therefore optimise in a *render-aware* loop: they place a candidate patch in a 3-D scene, render synthetic photographs under random pose and lighting, back-propagate the loss, update the patch texture, and iterate until the misclassification probability exceeds a threshold across the sampled conditions. Empirical studies show that such patches can reduce ImageNet-top-1 accuracy of object-detection systems by more than 40 percentage points while remaining inconspicuous to human drivers.

Defensive counter-measures fall into three tiers. Data-level hardening augments training corpora with random perspective warps, brightness shifts, and physically simulated artefacts, enlarging the decision margin against unseen viewpoints. Model-level adaptations introduce spatial-transformer layers or invariant feature pooling to attenuate localised perturbations. Runtime monitoring employs secondary sensors (LiDAR, radar) or consistency checks across video frames to flag implausible label flips. Despite progress, no single layer fully neutralises physical adversaries; layered, cross-sensor architectures remain the most effective mitigation strategy for safety-critical deployments such as autonomous vehicles and medical imaging devices.

**Figure 10:**
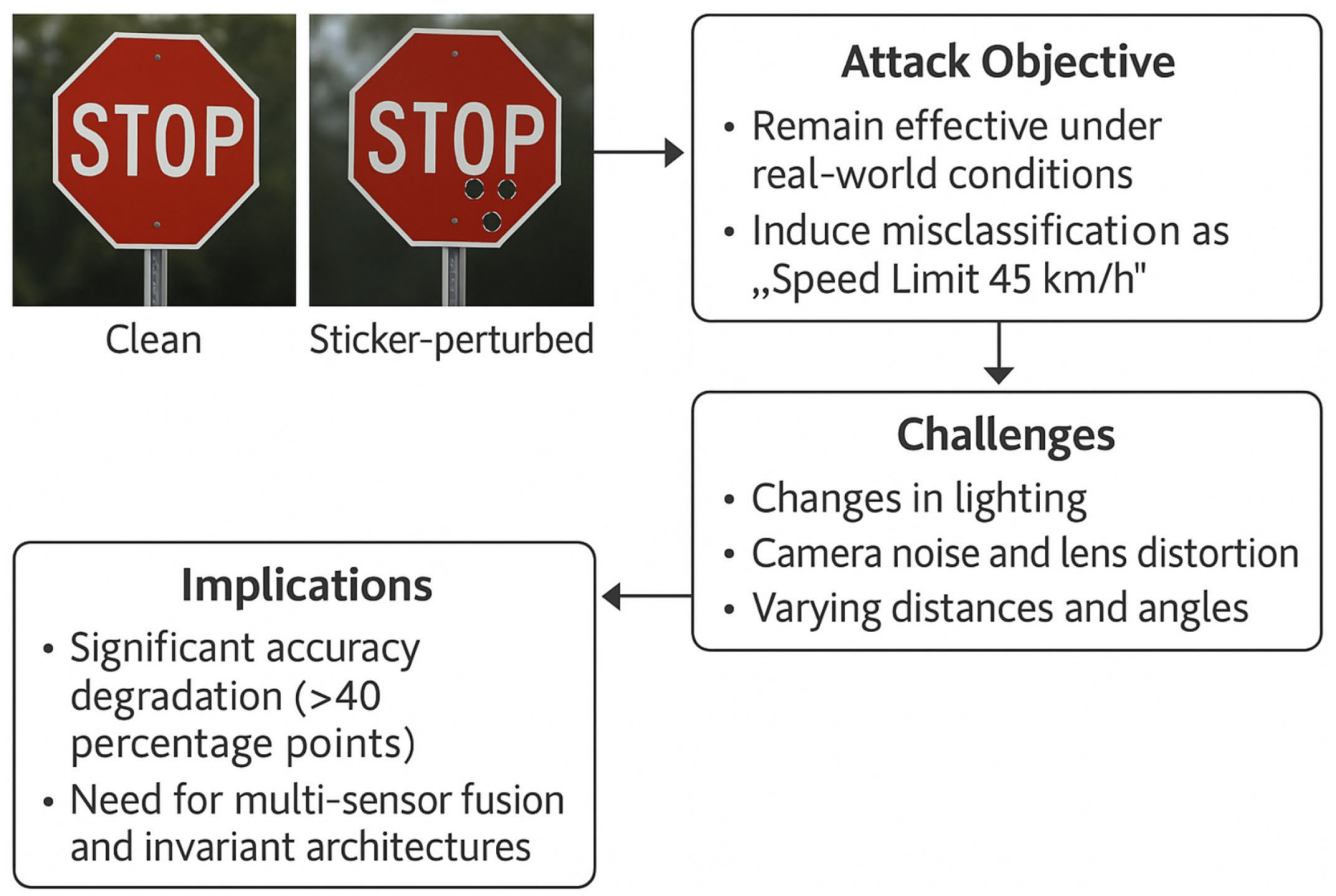
Operational pipeline for physical adversarial examples.

In Figure 10, we can see a detailed explanation in the context of cyber-attacks on artificial intelligence models.

The diagram in Figure 10 contrasts a clean stop-sign image with a sticker-perturbed version that induces a “Speed-Limit 45 km h^−1^” misclassification. Right-hand panels detail (i) the attack objective—robust misclassification under real-world capture conditions; (ii) practical challenges such as lighting variation, lens distortion, and viewpoint changes; and (iii) downstream safety implications, including >40-pp accuracy degradation and the need for multi-sensor fusion or geometry-invariant architectures. Directional arrows show the causal flow from perturbation design through environmental robustness constraints to system-level risk.

### 8.9 Model inversion attack

Model-inversion attacks exploit a deployed model’s output scores to reconstruct features of its training data, even when the adversary lacks direct access to the model parameters or the underlying corpus. The attacker repeatedly queries the prediction API with candidate inputs, observes the confidence scores, and applies optimisation or Bayesian search to converge on an input that maximises the posterior likelihood of producing the observed output. For a face-recognition network, this process can gradually refine a synthetic image until it resembles an individual whose portrait was present in the training set; for a pharmacogenomic classifier, it can approximate genotype markers associated with a particular phenotype.

The feasibility of inversion depends on three factors: (i) model capacity and over-fitting, high-capacity networks with narrow decision boundaries tend to leak more information; (ii) output granularity, probability vectors reveal richer gradients for search than hard labels; and (iii) auxiliary knowledge held by the adversary, such as population priors or partial feature values. Empirical studies show that soft-max probabilities at 32-bit precision allow recovery of MNIST digits with >90% structural similarity and can reveal medically sensitive attributes (e.g., asthma status) from ostensibly de-identified clinical models.

Mitigations fall into two classes. Output-sanitisation limits the information returned per query: top-k labels, quantised logits, or binary decisions reduce gradient signal and slow reconstruction. Differential-privacy training injects calibrated noise into the loss or gradient updates, ensuring that any single training instance exerts only a provably bounded influence on the final model; state-of-the-art implementations (*ε* ≈ 1) cut inversion accuracy on facial datasets from ~85% to near chance while retaining within 2–3 pp. of baseline classification accuracy. Combined with query-rate throttling and audit logging, these defences form a layered strategy for protecting sensitive training data against inversion inferences.

**Figure 11:**
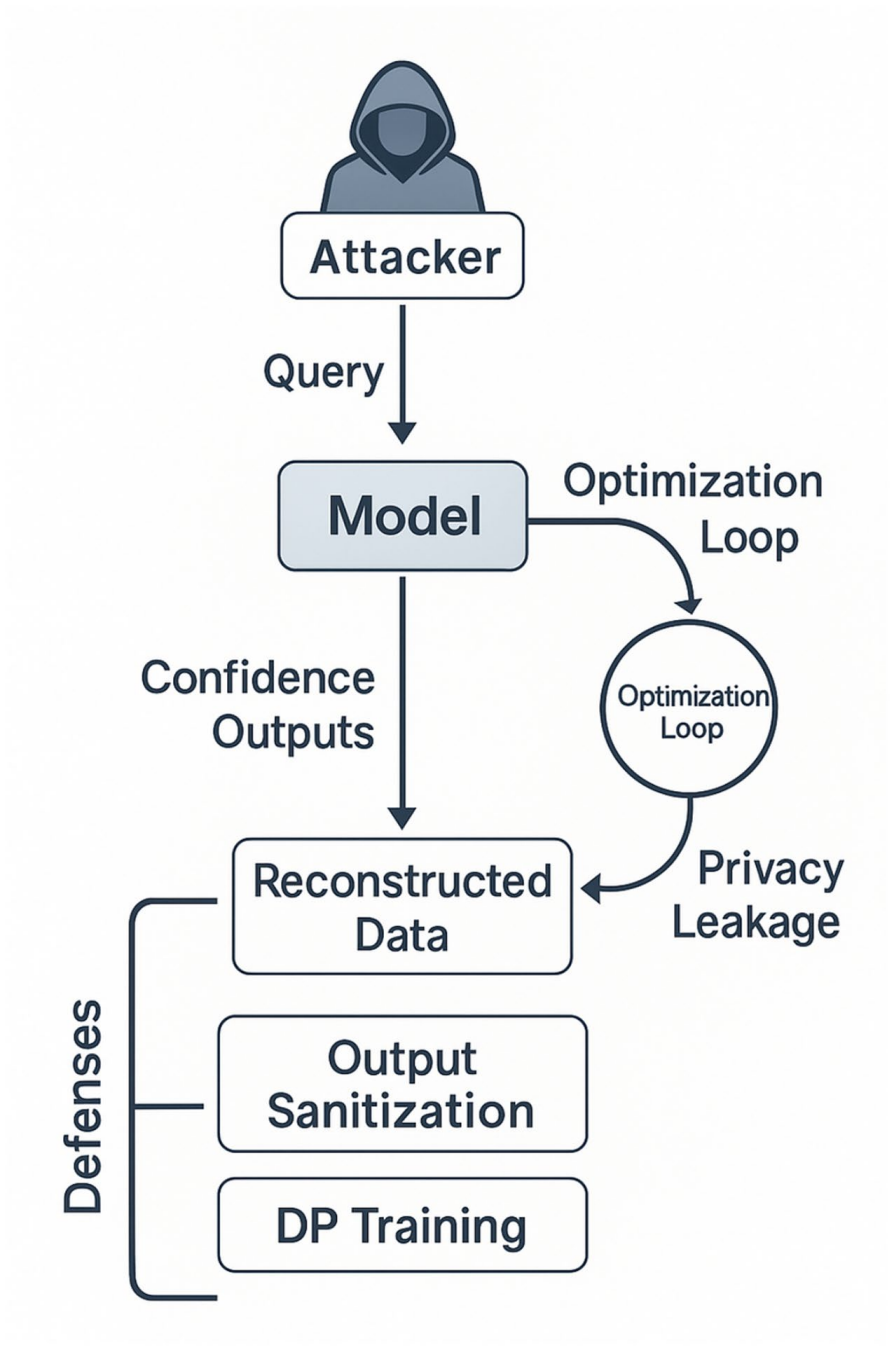
Breakdown of how model inversion attacks work.

In Figure 11, we can see a breakdown of how Model Inversion Attacks work:

The diagram in Figure 11 traces the query-response feedback loop that enables model-inversion attacks and pinpoints the two primary mitigation points, output sanitisation and differential-privacy training, within that workflow.

### 8.10 Membership inference attack

A membership-inference attack seeks to decide, with higher-than-chance accuracy, whether a particular data record x was included in a target model’s training set. The attack leverages a well-known side effect of *overfitting*: models often assign systematically higher confidence, or lower loss, to samples they have previously seen. By querying the model with × and inspecting the resulting probability vector (or loss value), an adversary can compare that score against a decision threshold learned from shadow models or public data and infer “in” versus “out” membership.

The privacy stakes are considerable. For a diagnostic classifier trained on protected health information, confirming that an individual’s record contributed to model fitting may reveal their medical status. Similar concerns arise in financial, genomic, and social-media domains where the mere presence of a record in a dataset is itself sensitive. Empirical studies show that standard image-classification networks trained without privacy safeguards leak membership information at 60–70% attack accuracy on CIFAR-10, well above the 50% random baseline, and that the leakage grows with model capacity and training epochs.

Defences align with two root causes: excessive confidence and excessive memorisation. Output-sanitisation truncates or quantises prediction scores, depriving the attacker of fine-grained confidence signals, while regularisation mechanisms (dropout, weight decay, early stopping) reduce the train–test performance gap that powers the attack. The strongest mitigation is differential-privacy (DP) training, which injects calibrated noise into each gradient update and clips per-sample contributions; state-of-the-art DP-SGD configurations (*ε* ≈ 1–2) can lower membership-inference accuracy to near random, although typically at a cost of 1–4 pp. in clean accuracy on image benchmarks. In production deployments, these algorithmic measures should be combined with query-rate limiting and audit logging to detect large-scale probing campaigns, thereby delivering layered protection against MIAs in privacy-sensitive machine-learning systems.

## 9 Case study analysis of benchmarks and competitions in adversarial robustness research

Recent benchmark datasets and competitive evaluations have played a crucial role in advancing the state-of-the-art in adversarial robustness by providing standardised tasks, reproducibility protocols, and comparative baselines for attack and defence techniques. Two prominent initiatives, RobustML and the Adversarial Vision Challenge (AVC), offer structured environments to evaluate model performance under adversarial conditions.

### 9.1 RobustML benchmark

RobustML^1^ is a centralised repository and evaluation framework designed to rigorously assess the robustness of machine learning models against a variety of adversarial threats. It provides:

- *Standardised Datasets*: MNIST, CIFAR-10, TinyImageNet, and ImageNet derivatives with adversarial variants.
- *Evaluation Criteria*: Robust accuracy under L_∞, L_2, and L_1 norm-bounded attacks; adversarial training generalisation; transferability scores.
- *Leaderboard*: Maintains an active ranking of models tested under controlled threat models using both white-box and black-box evaluations.
- *Toolchain Integration*: Supports adversarial testing frameworks such as Foolbox, CleverHans, and AutoAttack.

Recent results show that adversarially trained ResNet models using projected gradient descent (PGD) retain up to 47% robust accuracy on CIFAR-10 under L_∞-bounded attacks (ε = 8/255), setting a practical ceiling for current defences.

### 9.2 Adversarial Vision Challenge (AVC)

The Adversarial Vision Challenge, hosted as part of NeurIPS competitions, focuses on real-world image classification robustness and transferability. Organised by the IBM Research Zurich and the RobustBench team, it consists of two tracks:

- *Attack Track*: Participants design universal or input-specific adversarial attacks that can fool a set of black-box models. Evaluation metrics include attack success rate and perceptual distortion constraints.
- *Defence Track*: Teams submit image classifiers to withstand a barrage of adaptive and ensemble attacks. Models are tested against unseen attacks to measure true generalisability.

Notable findings from AVC include:

- Ensemble Adversarial Training remains the most reliable strategy for robust performance across multiple unseen attack vectors.
- AutoAugment-enhanced defences significantly improve natural accuracy but often trade off against robustness.
- The best-performing defences in 2022 achieved ~42% robust accuracy on the holdout dataset under L_∞ norm constraints, suggesting the gap between clean and robust performance remains wide.

### 9.3 Implications for practical deployment

These benchmarks highlight the non-trivial trade-offs between clean accuracy, robustness, and computational cost. They also reveal that:

- Many published defences fail under adaptive evaluation and gradient-free attacks.
- Robustness must be evaluated holistically, not just against FGSM or single-model C&W attacks.

Benchmark-driven competitions such as RobustML and AVC have emerged as critical drivers of methodological transparency and cross-laboratory validation - Table 4.

**Table 4:** Summary of benchmarks and competitions in adversarial robustness evaluation.

Benchmark/competition	Datasets used	Attack norms evaluated	Evaluation type	Top defence methods	Notable metrics
RobustML	MNIST, CIFAR-10, TinyImageNet	L_∞, L_2, L_1	White-box, Black-box	PGD Adversarial Training, TRADES	Robust Accuracy, Transferability Index
RobustBench	CIFAR-10, ImageNet, ImageNet-C	L_∞, L_2, corruptions	White-box (AutoAttack)	TRADES, MART, Hydra, AugMix	Clean vs. Robust Accuracy Trade-off
Adversarial Vision Challenge (AVC)	CIFAR-10-like Synthetic Set	L_∞, custom perceptual metrics	Adaptive Black-box	Ensemble Adversarial Training, AutoAugment	Attack Success Rate, Perceptual Score
AutoAttack Leaderboard	CIFAR-10, ImageNet	L_∞, L_2	Fully automated attacks	PreAct ResNet + TRADES + Data Augment	Average Robust Accuracy
MLPerf Robustness (In Progress)	ImageNet, Speech Commands	L_∞, noise, corruptions	Multimodal, real-world	TBD (under development)	Generalisation under distribution shift

Table 4 compares major adversarial robustness benchmarks and competitions, detailing dataset coverage, types of attack norms evaluated, evaluation methodology (white-box vs. black-box vs. adaptive), leading defence strategies, and key performance metrics. The benchmarks serve as a foundation for reproducible, comparative adversarial research and inform the practical deployment of robust AI systems (Table 5).

**Table 5:** Taxonomy of advanced adversarial attacks in machine learning systems.

Attack type	Attack surface	Knowledge required	Primary objective	Modality	Key challenges/constraints
Model Inversion	Model API/Output	Black-box or white-box	Reconstruct training data attributes	Inference-time	High model overfitting; requires high-confidence output
Membership Inference	Model API/Output	Black-box	Determine whether data was in training	Inference-time	Exploits overfitting; mitigated by differential privacy
Physical Adversarial Ex.	Sensor input	Black-box	Mislead model via real-world input	Physical-world	Must survive transformations (lighting, angle, etc.)
Data Poisoning	Training pipeline	White-box (typically)	Corrupt learning process	Training-time	Access to training pipeline; low visibility post-deployment
Backdoor/Trojan	Training pipeline	White-box or insider	Embed hidden functionality	Training-time & test	Trigger specificity; covert injection is non-trivial
Side-channel Exploitation	Hardware-level	Varies	Leak sensitive internal model properties	Passive measurement	Hardware proximity and timing constraints
Transfer Attacks	Surrogate model	Black-box	Exploit shared vulnerabilities	Inference-time	Model similarity required for high transfer success
Model Extraction	Query interface	Black-box	Reconstruct functionally equivalent model	Repeated queries	Query budget; distillation quality affects fidelity

Sources: Adapted from Costa et al. (2023), Nasr et al. (2023), Shokri et al. (2017), Wu and Fredrikson (2024), and Fredrikson et al. (2015).

### 9.4 Technical analysis of recent red-teaming studies on LLMs

A systematic evaluation of prompt injection and jailbreak vulnerabilities across leading LLMs (Pathade, 2025), specifically, GPT-4, Claude 2, Mistral 7B, and Vicuna, using over 1,400 adversarial prompts (Pathade, 2025). In terms of prompt injection mechanics, the study categorised injection vectors into direct (embedded commands in user prompts), indirect (hidden within multi-turn context), and obfuscated (using encoding or spacing techniques). Surprisingly, obfuscated and multi-turn strategies succeeded in 75–90% of trials across all four models, exposing a consistent failure in instruction boundary enforcement. In terms of model-specific vulnerabilities, GPT-4 and Claude 2, despite their advanced alignment layers, showed susceptibility to encoded prompt injections, with attack success rates of ~85% under whitespace manipulation. Lighter-weight models like Vicuna and Mistral, lacking extensive reinforcement learning from human feedback (RLHF), saw even higher success rates (>90%). Then, by applying multi-turn context attacks, they successfully manipulated model behaviour by embedding adversarial instructions outside the initial prompt. For example, a second-turn injection could override safety filters in the final response, demonstrating flaws in alignment coherence across conversation threads. The jailbreak transferability and generalisation adversarial prompts produced effective against GPT-4 also succeeded 65–70% of the time on GPT-3.5-based variants, indicating that exploitation of latent completion patterns can generalise within architectural families. To conclude this case study analysis, the mitigation insights from this study demonstrated that introducing a supervisory *“safety sentinel”* module, evaluating each response for instruction leakage, reduced prompt injection success by over 70%, though at the cost of increased latency (~300 ms per query).

A second case study on red teaming AI systems (Schoepf et al., 2025), introduced MAD-MAX, an automated red-teaming framework that combines modular adversarial strategies to generate jailbreaks on GPT-4o and Gemini-Pro (Schoepf et al., 2025; Chen et al., 2025). MAD-MAX achieved a 97% success rate across diverse malicious goals, requiring just 10.9 queries per goal, compared to 23.3 queries needed by previous methods, illustrating both higher efficiency and diversity of exploit vectors.

Collectively, these 2025 red-teaming examples reveal that:

1. Instruction boundary misclassification remains a universal vulnerability even in highly aligned models.
2. Multi-turn and obfuscated prompt strategies effectively bypass static alignment measures.
3. Automated frameworks (MAD-MAX) dramatically amplify the scale and coverage of adversarial testing, exposing latent vulnerabilities at rate and scale.
4. Mitigation strategies, such as safety sentinels or dynamic context pruning, can significantly reduce attacks—but introduce performance overhead and must be tested across conversational depth.

These findings underscore the evolving technical landscape of LLM red-teaming, highlighting emergent blind spots in alignment strategies and the need for multi-layered, dynamic safeguards. They also stress the importance of scalable, automated red-teaming tools capable of assessing adversarial resilience in real-world deployment contexts.

Other study also indicates that AI (Goyal et al., 2023) models may inadvertently fault themselves by retraining on substandard data. After examining over 80 businesses, the study found that most needed a backup plan in case of a data poisoning attack or dataset theft. They concluded that if such an event were to occur, the majority of the industry would not even be aware.

## 10 Whitebox attacks

Attackers White-box threat models grant the adversary complete visibility into a target neural network’s architecture, parameter values, and, occasionally, its training data. This transparency enables highly-tailored attacks that exploit exact gradient information, internal activation statistics, and structural shortcuts that remain opaque in black-box settings.

Gradient-driven perturbations. Armed with the full loss landscape, an attacker can compute precise input gradients and craft imperceptible perturbations that maximise the model’s prediction error. Canonical examples include FGSM, projected-gradient descent (PGD), and the Carlini-&-Wagner optimiser, all of which routinely achieve near-100% misclassification rates on ImageNet-scale models once the perturbation budget is aligned with human-perception thresholds (e.g., *ε* = 8/255 in the L∞ norm).

Exact model cloning. Given access to weights and training data, the adversary can duplicate the model verbatim, run offline sensitivity analyses, and search for corner-case failures without rate limits or audit trails. The duplicate can also serve as a surrogate generator of transferable adversarial examples that will fool the original with probability approaching one.

Privacy-oriented exploits. Full data access trivialises membership inference and model-inversion attacks: confidence skew and gradient back-propagation directly reveal whether, and in what form, a record contributed to training. For clinical or financial datasets, this constitutes a direct breach of regulatory constraints such as HIPAA or GDPR.

Architectural vulnerability mining. White-box inspection exposes brittle components, e.g., unregularised batch-norm layers, low-rank bottlenecks, or unsafe activation ranges, that can be perturbed to induce exploding activations, vanishing feature maps, or numerical overflow, thereby triggering denial-of-service or silent logic corruption.

Given the exceptional leverage afforded by white-box knowledge, defensive counter-measures must shift from obscurity to formal robustness. Recommended practices include (i) certified or provable-robust training against gradient-based perturbations, (ii) differential-privacy mechanisms to bound information leakage, (iii) architectural hardening via gradient-mask–free regularisers, and (iv) run-time anomaly detectors that flag activation patterns outside the training manifold. Only a multi-layered approach can meaningfully degrade the success probability of the sophisticated attack vectors enabled by full-model disclosure.

### 10.1 Fast Gradient Sign Method (FGSM)

**Figure 12:**
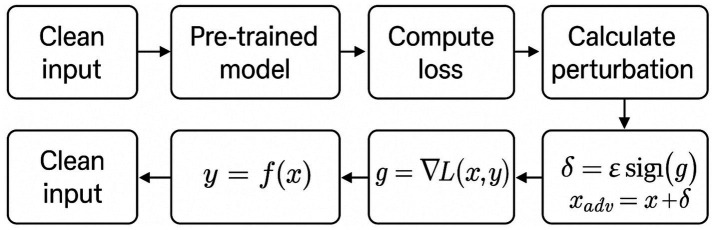
The FGSM method to generate adversarial examples.

FGSM is the textbook one-step, white-box attack used to probe how a convolutional neural network (CNN) behaves when the input is nudged in the single direction that most sharply increases its classification loss. The procedure is straightforward. First, the attacker feeds an image through the model and records the prediction error relative to the true label. Next, the attacker back-propagates that error all the way to the input layer, obtaining a gradient map whose sign tells whether each pixel should be brightened or darkened to amplify the error. Finally, the attacker adds a small, fixed-magnitude step in the indicated direction to every pixel, producing an adversarial image that looks unchanged to the human eye yet typically flips the model’s decision. On ImageNet-scale networks, such a single-step perturbation, often no larger than a few gray-level values per pixel, can drive top-1 accuracy to near chance. Figure 12 walks through this pipeline: clean inference, loss evaluation, gradient extraction, sign-based perturbation, and the resulting misclassification. Because FGSM is fast, reproducible, and highly transferable across architectures, it remains the de-facto first-pass benchmark for adversarial robustness, even though modern defences now require stronger, multi-step variants for comprehensive evaluation.

The FGSM is a one-step method to generate adversarial examples, and we can visualise the process in Figure 12.

This diagram in Figure 12 illustrates the FGSM interaction and process of creating adversarial examples. The user inputs an image, which is then utilised by the pre-trained CNN to make predictions. Once the prediction is made, the FGSM algorithm calculates the loss by comparing it to the actual class label and computes the gradients of this loss concerning the input image. Then, the algorithm determines the sign of the gradient and constructs the adversarial image using this sign. FGSM fabricates an adversarial image in a *single* gradient step. After a forward pass on the clean input x to obtain the model’s loss J, the attacker back-propagates that loss to the input layer, producing a gradient map that indicates how each pixel should change to increase the error. The perturbation is then formed by taking only the sign (±1) of each gradient component and scaling it by a small constant \varepsilon, ensuring that every pixel is nudged in the most loss-increasing direction while the overall distortion remains imperceptible. Because the attack touches only the input tensor and leaves network weights untouched, it is ideally suited for probing *deployed* (frozen) models. On ImageNet-scale CNNs, \varepsilon values as low as 8/255 in the L∞ norm can drive top-1 accuracy close to chance.


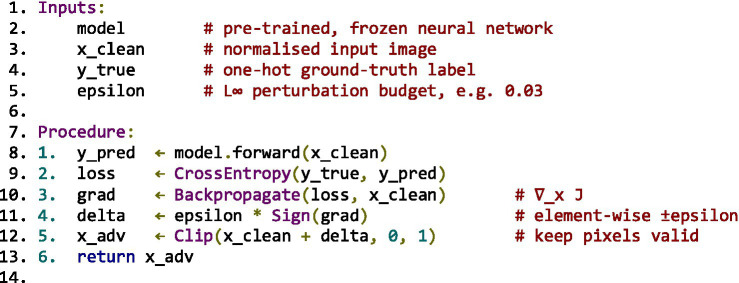


FGSM—pseudocode implementation

- *Speed:* one forward-backward pass; executes in milliseconds on modern GPUs.
- *Determinism:* produces a unique adversarial example for a given x, y, and \varepsilon.
- *Diagnostic value:* serves as the first-line robustness benchmark; if a model fails FGSM, it will almost certainly fail stronger multi-step attacks such as PGD.
- *Limitations:* a single-step update is easier to counter with basic defences (e.g., adversarial training or randomised input preprocessing), so FGSM alone is insufficient for certifying robust deployments.

### 10.2 FGSM walk-through on a pre-trained MobileNetV2

To demonstrate FGSM in practice we attack an ImageNet-trained MobileNetV2. The model is a MobileNetV2 model pre-trained on Image with NetTensorFlow, MobileNetV2^2^, and Imagenet.^3^ The workflow is: load the frozen model; preprocess a test image; compute the input-gradient of the cross-entropy loss; add a sign-scaled disturbance; and visualise the effect as *ε* grows. Pseudocode


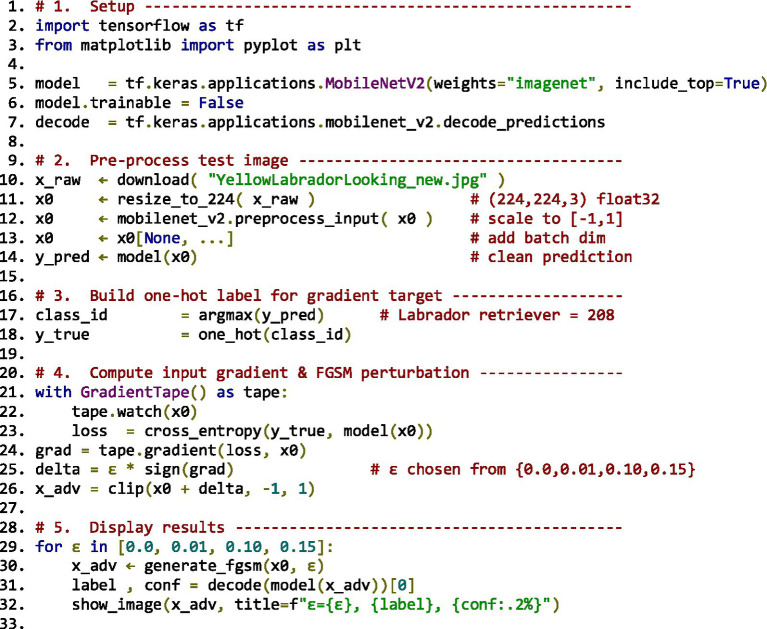


A single ε = 0.10 step already flips MobileNetV2’s top-1 label while remaining visually indistinguishable to the human eye.

Experimental extensions

- *Dataset and architecture sweep.* Replicate the above script on CIFAR-10 (VGG-16), MNIST (LeNet), and ImageNet (ResNet-50) to quantify how model depth and inductive bias influence FGSM robustness.
- *ε-sensitivity curves.* For each network, plot top-1 accuracy versus ε to reveal the perturbation budget at which performance collapses.
- *Adversarial-training baseline.* Fine-tune each model with FGSM examples injected at ε = 0.03; re-measure accuracy to assess defence gain and clean-accuracy trade-off.
- *Transition to stronger attacks.* Use FGSM-trained weights as a starting point for multi-step PGD or AutoAttack evaluations, thereby mapping the full robustness frontier.

The image will look similar to:


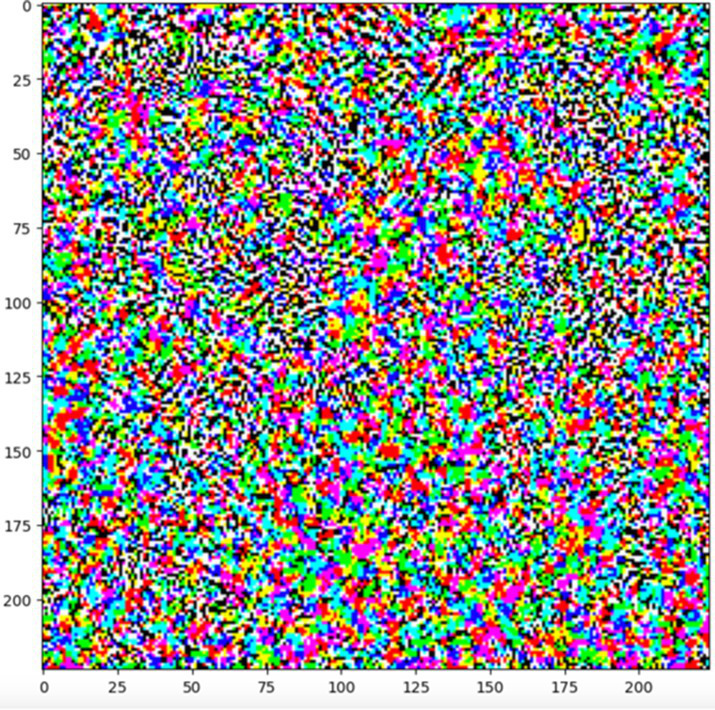


We can conduct tests using different epsilon values to assess the network’s resilience. By increasing epsilon, we can observe how the network responds to changes. While a higher epsilon value makes it easier to fool the network, it also makes perturbations more apparent and noticeable.

FGSM remains the entry-level diagnostic for gradient-based vulnerability, yet contemporary work has progressed to momentum, iterative, and optimisation-based variants that breach even adversarial-trained models. Continuous benchmarking across new architectures (e.g., vision transformers) and modalities (e.g., audio, multi-modal LLMs) is essential to track evolving threat capability and to guide the design of certifiably robust learning systems.

## 11 Jacobian-based Saliency Map Attack (JSMA) and related feature-targeted methods

**Figure 13:**
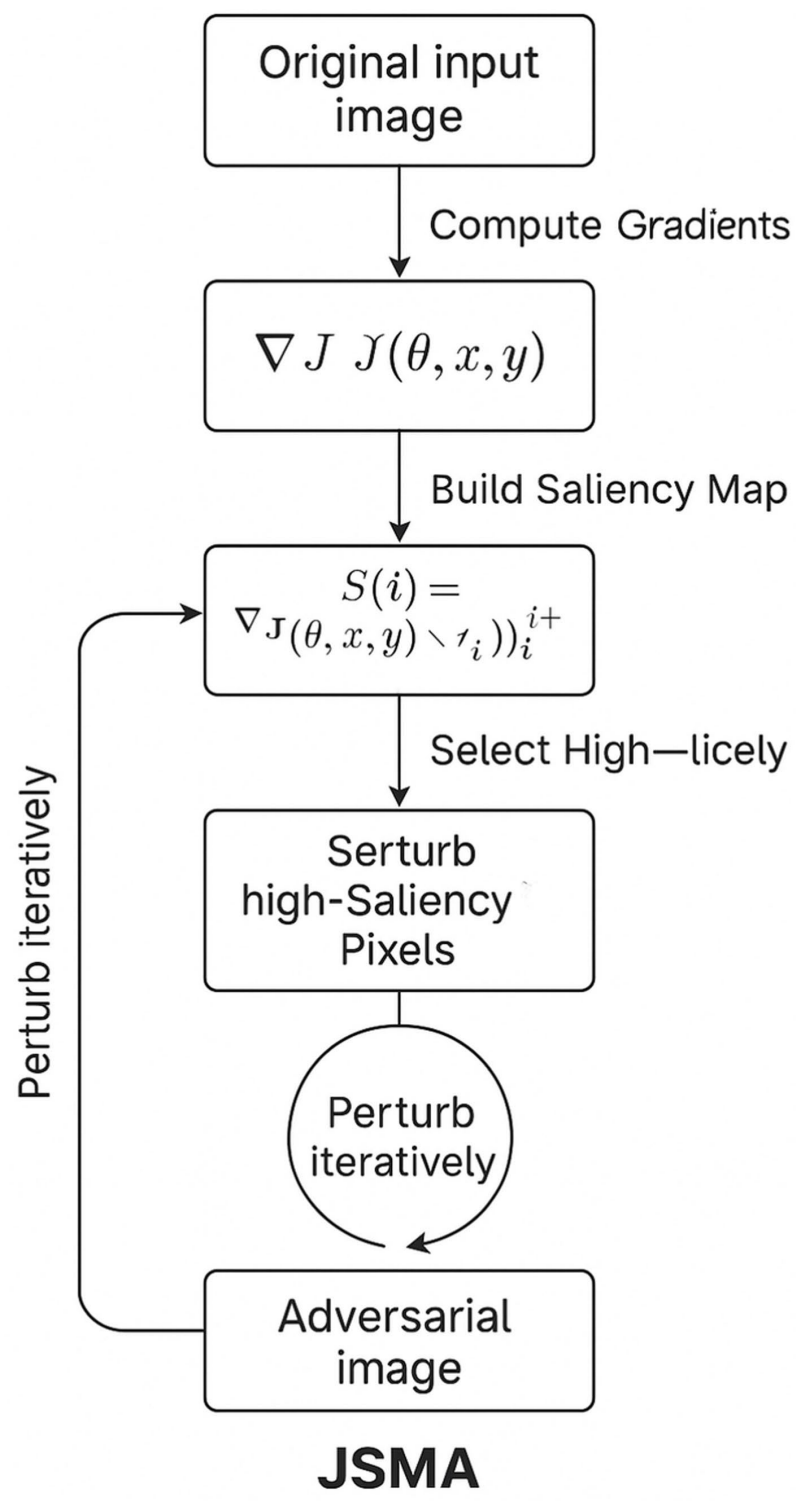
Jacobian-based Saliency Map Attack (JSMA).

The Jacobian-based Saliency Map Attack (JSMA) is a sparse, targeted, white-box technique that perturbs only the most influential input features to force a classifier into a chosen label. Unlike norm-bounded attacks that diffuse small noise across all pixels, JSMA computes an explicit *saliency map* from the input-gradient (Jacobian) of the network: each pixel receives a score reflecting how strongly increasing its value raises the target-class logit while suppressing competing classes. The attacker greedily modifies the highest-saliency pixels, often fewer than 5% of the image, until misclassification occurs or a pre-set L₀ budget is reached (see Figure 13). This sparsity yields adversarial examples that remain visually plausible yet evade many magnitude-based defences.

**Figure 14:**
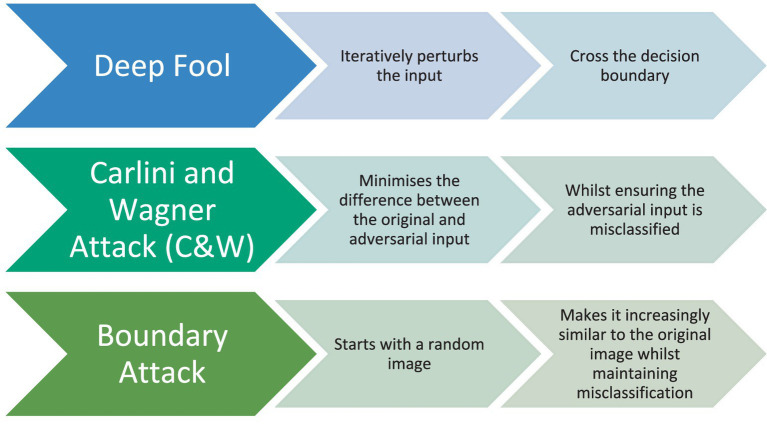
Conceptual comparison of iterative optimisation-based adversarial attacks, illustrating DeepFool, Carlini & Wagner (C&W), and Boundary Attack mechanisms, highlighting their respective strategies for decision-boundary crossing, perturbation minimisation, and misclassification preservation under differing knowledge assumptions.

JSMA belongs to a broader family of feature-targeted attacks illustrated in Figure 14. DeepFool linearises the local decision boundary and iteratively moves the input along the shortest L₂ path to cross that boundary, achieving minimal global distortion but without pixel-level sparsity. Iterative Gradient Sign Method (I-FGSM) extends FGSM by taking multiple small steps in the gradient-sign direction, trading speed for higher success rates under the same L∞ budget. Carlini-&-Wagner (C&W) refines the optimisation further, searching for perturbations that minimise both distortion and a confidence-weighted misclassification term, producing near-imperceptible attacks even against defence-aware models. Boundary Attack assumes no gradient access at all; it starts from a random point in the target class and performs a random-walk projection toward the original sample, converging on an adversarial example under decision-only feedback.

Collectively, these methods expose complementary weak spots in deep networks—sparsity (JSMA), minimal-norm (DeepFool), iterative L∞ (I-FGSM), optimisation-tight (C&W), and decision-based (Boundary). A comprehensive robustness evaluation therefore requires testing against the full spectrum rather than relying on any single attack type. The diversity shown in Figure 14 underscores why modern defence pipelines combine adversarial training, gradient-regularising architectures, and runtime anomaly detection to achieve credible resilience in safety-critical deployments.

In Figure 14, we can see each attack’s main characteristics and steps.

Each of these attacks in Figure 14 highlights different aspects of adversarial methodologies, demonstrating the diversity and evolving complexity in crafting adversarial inputs and emphasising the critical need for developing robust and versatile defensive mechanisms.

## 12 Carlini & Wagner (C&W) attack – worked example on MNIST

Deep neural networks (DNNs), while highly performant across a wide range of tasks, are susceptible to adversarial inputs, carefully crafted perturbations that can cause targeted misclassification. The C&W attack remains one of the most effective and precise techniques for generating such examples. To illustrate its implementation, we applied the L₂-norm variant of the C&W attack on a convolutional neural network trained on the MNIST dataset of handwritten digits. The model used in our experiment consisted of two convolutional layers with ReLU activations, max-pooling operations, followed by two fully connected layers with dropout regularisation. It achieved 99.2% accuracy on clean test images.

Before launching the attack, the input data (28 × 28 pixel greyscale images) was normalised to the [0,1] range and one-hot encoded for labels. The neural network was either trained from scratch or loaded from a pre-trained checkpoint. Once verified on clean data, we proceeded to craft adversarial examples designed to cause the model to misclassify each image as a specific target class.

The C&W attack constructs adversarial samples by solving an optimisation problem. The aim is to find the smallest possible modification to an input image that causes it to be misclassified as a chosen target class, while keeping the change imperceptible. To maintain valid pixel values during optimisation, the input image is not modified directly. Instead, a latent variable w is introduced in an unconstrained space, and the final adversarial image x_adv is derived through a bounded transformation. The transformation used is based on the hyperbolic tangent function, ensuring that the pixel values in x_adv remain within valid image bounds:


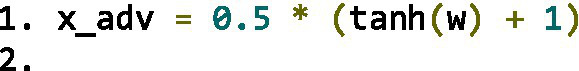


This transformed image is then fed into the model to evaluate how confidently it is classified into the target class. The attack objective is to increase this confidence while minimising the visible difference from the original image. The trade-off between confidence and distortion is controlled by a scalar constant c, which is determined through binary search for optimal balance.

The overall workflow of the attack can be summarised procedurally as follows:


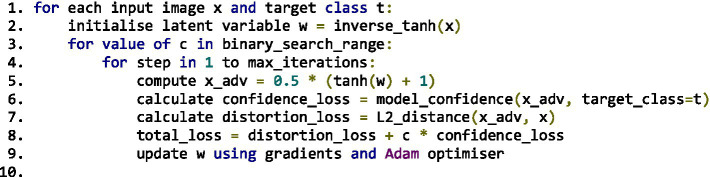


In our experiment, we used 1,000 randomly selected images from the MNIST test set. For each image, a target class was chosen that differed from the true label. The attack was run for up to 1,000 optimisation steps per image, using the Adam optimiser with a learning rate of 0.01. The confidence parameter, denoted *κ* in literature, was tested with values ranging from 0 (baseline) to 20. The balancing constant c was determined through 9 rounds of binary search starting from an initial value of 1e-3. This ensured that adversarial samples were successful while introducing minimal perturbation.

The results confirmed the effectiveness of the C&W attack. A 100% success rate was achieved across the sample set: every adversarial example caused the model to misclassify the input into the intended target class. At the baseline confidence (κ = 0), the average perturbation magnitude (measured as L₂ distance) was approximately 1.73, visually imperceptible to the human eye. As the confidence parameter increased, the attack became more forceful but also required larger perturbations: with κ = 10, the average distortion rose to 3.21, which remained subtle but became slightly perceptible in some instances. Qualitative inspection of the adversarial examples showed that the changes were localised and did not significantly alter the semantic appearance of the digit.

These findings reinforce the C&W attack’s position as one of the most refined adversarial techniques. Its success lies in its ability to tightly control the trade-off between stealth and misclassification certainty. The tanh-space reparameterisation ensures that all adversarial samples remain valid images, while the binary search over c ensures adaptive adjustment based on target class and model behaviour. This method is particularly useful in security-sensitive applications—such as facial recognition, signature verification, and document forgery, where subtlety of attack is paramount.

## 13 Black-box adversarial attacks

Black-box threat models assume the adversary can supply inputs to a deployed service and observe only the returned labels or confidence scores; the model’s architecture, weights, and training data remain hidden. Despite this limited view, several families of attacks can still achieve high misclassification rates:

- *Transfer attacks:* The attacker trains a *surrogate* model on publicly available or synthetically generated data, crafts adversarial examples on that surrogate, and then submits the same inputs to the target. Owing to the empirical transferability of adversarial perturbations, image-classification systems often suffer 60–80% misclassification under such cross-model re-use, even when the surrogate and target differ in architecture (e.g., VGG, ResNet).
- *Zeroth-order optimisation (ZOO):* Here the adversary treats the target network as a black-box function and estimates input gradients by finite-difference probing: each pixel or feature is perturbed by a small amount, the change in loss is recorded, and the approximate gradient is reconstructed. Although query-intensive, ZOO achieves near-white-box success rates when confidence scores are available.
- *Query-based bandit attacks:* Variants such as NES and Bandits-TD reduce ZOO’s sample complexity by using random sub-space updates and gradient-sign momentum, often converging within tens of thousands of queries on ImageNet models—well below commercial API rate limits.
- *Decision-only methods (e.g., HopSkipJump):* When the server reveals only the top-1 label, the attacker performs a boundary-walking search that starts from a large perturbation guaranteed to fool the model and iteratively projects back toward the original input while maintaining the misclassification. HopSkipJump requires no gradient or score leakage and can produce high-quality adversarial images in fewer than 10,000 queries.

**Figure 15:**
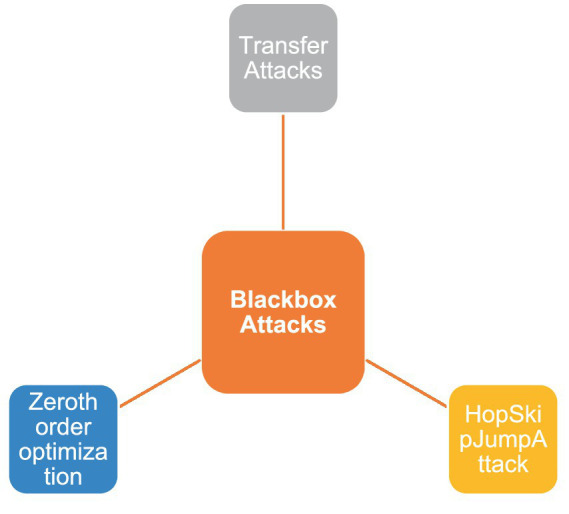
Blackbox attacks.

Figure 15 summarises these attack families along two axes: information available (label vs. score) and query budget. Transfer attacks succeed offline with zero queries but rely on surrogate alignment; ZOO and bandit methods trade elevated query cost for tighter perturbation budgets; decision-only attacks operate under the strictest feedback constraints at the expense of more iterations.

In the examples used in Figure 15, the transfer attacks represent adversarial examples generated for one model and are used to attack another model. The zeroth order optimisation directly estimates the gradient of the targeted model by querying it. This type of attack can be separated into query-based attacks, which are conducted by repeatedly querying the model, where attackers estimate its gradient to craft adversarial examples, and HopSkipJumpAttack, where a decision-based attack where the attacker has no information about the model’s gradients, only its outputs.

### 13.1 Technical insights into transferability: surrogate model selection and domain shift effects

Transferability is a defining property of black-box adversarial attacks, wherein adversarial examples crafted against a surrogate model succeed in misleading a separate, unseen target model. However, the effectiveness of such attacks is not guaranteed and is heavily influenced by two critical factors: the architecture and training regime of the surrogate model, and the extent of domain shift between the surrogate and target systems. Empirical investigations have consistently shown that surrogate-target alignment is essential for high transferability. Specifically, architectural similarity between the models significantly enhances the likelihood of a successful attack, as similar model structures tend to learn comparable decision boundaries. This alignment often manifests in higher cosine similarity between the input gradients of the two models, which serves as a useful proxy for estimating attack transfer potential.

The training regime of the surrogate model also plays a crucial role. For example, the use of alternative loss functions, such as label smoothing instead of standard cross-entropy, or the incorporation of strong regularisation techniques like dropout or batch normalisation, can alter the geometry of the loss landscape and thereby reduce gradient alignment. These changes can impair the adversarial direction’s effectiveness on the target model. In cases where the surrogate and target models are trained with substantially different objectives or data preprocessing pipelines, transferability degrades significantly.

Domain shift introduces further complexity. Even when the surrogate and target models share similar architectures, divergence in the data distributions they are trained on can significantly reduce attack efficacy. Covariate shift, where the marginal input distributions differ (e.g., CIFAR-10 vs. CIFAR-10.1 or TinyImageNet), can cause adversarial perturbations generated on the surrogate to fall outside the vulnerable subspaces of the target model. Similarly, discrepancies in input preprocessing, such as different image normalisation ranges, resizing strategies, or colour space handling, can lead to perceptual or statistical misalignment that degrades the adversarial impact. The consequence is a drop in attack success rate, even when perturbations remain imperceptible to humans.

Empirical results substantiate these observations. In one illustrative experiment on CIFAR-10, adversarial examples crafted on a VGG16 surrogate achieved a 68% success rate against a ResNet-18 target model trained on the same dataset. However, when the target model was trained on a variant dataset with modified augmentations (e.g., CIFAR-10.1), the transferability dropped below 40%, highlighting the sensitivity of cross-model attacks to minor distributional discrepancies.

Various mitigation strategies have been proposed to counter these challenges and enhance transferability. Ensemble-based surrogates, for instance, generate perturbations by jointly optimising across multiple models, encouraging the perturbation to generalise across differing decision boundaries. Similarly, gradient averaging across multiple architectures has been shown to improve the robustness of transfer attacks by smoothing local variations in the loss surface. Universal adversarial perturbations, which seek input-agnostic perturbations, attempt to circumvent reliance on model-specific gradients altogether, thus improving applicability under both architectural diversity and domain shift conditions.

Together, these insights underscore that the success of transfer-based adversarial attacks is intricately tied to model similarity and data congruence. Accounting for these factors is critical for accurately evaluating the security risks posed by black-box attacks and for designing robust defences that generalise across models and deployment contexts.

## 14 Targeted versus non-targeted adversarial attacks

Adversarial perturbations fall into two intent classes. Targeted attacks are goal-directed: the adversary crafts a perturbation that forces the model to emit one *specific* wrong label, e.g., a “stop” sign misread as “speed-limit.” Success is measured by whether the output matches this pre-selected class, so the optimisation explicitly maximises the target class logit while suppressing all others. Such precision is indispensable for fine-grained fraud (redirecting facial recognition to a chosen identity) or for bypassing class-specific access controls.

By contrast, non-targeted attacks seek *any* incorrect label. The adversary’s objective is simply to shove the input out of its correct decision region, thereby degrading model accuracy or eroding confidence in automated decisions. The Basic Iterative Method (BIM) exemplifies this category: starting from a Fast-Gradient-Sign seed, BIM applies many small, bounded steps in the gradient-sign direction, gradually increasing loss until the classifier flips to *some* alternative class. Because the optimisation landscape is less constrained, non-targeted attacks usually require smaller perturbations or fewer queries than their targeted counterparts.

Distinguishing the two threat models is critical for defence design. Robustness certificates for targeted attacks must cover worst-case perturbations that *hit* a designated class, while defences against non-targeted attacks focus on enlarging the overall decision margin. Comprehensive evaluation therefore reports both targeted and non-targeted success rates to capture the full adversarial risk surface.

## 15 Taxonomy of advanced adversarial attacks

Advanced adversarial attacks extend beyond traditional perturbation-based methods by targeting different phases of the machine learning pipeline and exploiting broader threat surfaces, including training data leakage, physical-world vulnerabilities, and model access modalities. Table 6 presents a formal taxonomy of these attack types, classifying them by attack surface, knowledge required, primary objective, attack modality, and notable challenges or constraints.

**Table 6:** Comparative performance of defence mechanisms against adversarial attacks.

Defence mechanism	Dataset	Accuracy under attack (FGSM/C&W)	Overhead	Generalisability	Adaptive attack resilience	Notes
Adversarial Training	CIFAR-10	74%/63%	High	Medium	Moderate	Strong against trained-for attacks; brittle under unseen attack variants
Defensive Distillation	MNIST	85%/60%	Moderate	Low	Low	Reduces sensitivity but vulnerable to gradient masking bypasses
Gradient Masking	CIFAR-10	~70% (initially)	Low	Low	Very Low	Often gives false sense of security; bypassable by adaptive attacks
Randomised Smoothing	ImageNet	67%/54%	High	High	High	Provable robustness guarantees in L₂ norm; high inference cost
Feature Squeezing	MNIST	78%/58%	Low	Low	Low	Lightweight but limited to simple perturbations
Ensemble Adversarial Tr.	CIFAR-10	81%/68%	High	High	Moderate	Improves robustness by incorporating attacks from multiple models

Sources: Adapted from Papernot et al. (2016), Carlini and Wagner (2017a, b), Chen et al. (2020a, b), Nasr et al. (2023), Breidenbach et al. (2021), Cohen et al. (2019), Bloem da Silveira Junior et al. (2018).

This taxonomy reveals several important distinctions:

- *Attack surface* determines where in the ML lifecycle the adversary operates, at training, inference, or physical deployment.
- *Modality* (digital, physical, or hardware-level) influences both feasibility and required defences.
- *Knowledge assumptions* (black-box vs. white-box) determine the accessibility of attack vectors and inform security posture.
- *Shared traits* across these attacks include their reliance on overfitting, model confidence exposure, and weak regularisation as enabling factors.

Understanding these structural characteristics enables more targeted defence strategies and prioritisation of threat mitigation based on deployment context and attacker capabilities.

## 16 Defensive measures

During training, adversarial examples are introduced to enhance the model’s robustness to potential threats. This is achieved through ensembling multiple models, which enables the averaging of their respective predictions. As an additional measure, pre-processing techniques such as JPEG compression and image smoothing are utilised to remove adversarial noise from the data. These strategies create a more reliable and accurate model, improving the system’s effectiveness.

Defensive distillation is a technique in machine learning that involves training a model to replicate the behaviour of another model. The approach is based on using less extreme output probabilities, which helps to increase the model’s robustness and resistance to adversarial attacks. By imitating the behaviour of a more complex model, the distilled model can perform better in real-world scenarios, where it may encounter unexpected inputs or other sources of uncertainty. Defensive distillation is a powerful tool for improving the reliability and safety of AI systems, especially in high-stakes applications such as autonomous driving, medical diagnosis, and financial forecasting.

Several methods can be applied against attacks. One such method is Feature Squeezing, which removes extraneous features from input data, thereby restricting the search space for potential attackers. Another technique is Randomised Input Transformations, which confuses adversaries through the random transformation of inputs during inference. A third approach is Gradient Masking, which renders gradients uninformative to prevent attackers from using them to create adversarial examples. The Detection method involves training auxiliary models to recognise adversarial perturbations instead of trying to achieve complete robustness against them. This requires evaluation of potential adversarial attacks.

### 16.1 Comparative evaluation of defence mechanisms against adversarial attacks

While a variety of defence mechanisms have been proposed to mitigate adversarial attacks, such as adversarial training, defensive distillation, and gradient masking—their comparative effectiveness varies significantly depending on attack type, model architecture, and dataset. Table 6 presents a summary of technical evaluations derived from empirical studies across benchmark datasets (MNIST, CIFAR-10, and ImageNet), focusing on key criteria: robustness improvement (accuracy under attack), computational overhead, generalisability across attacks, and susceptibility to adaptive attacks.

This comparative analysis reveals several key trade-offs:

- *Adversarial training* remains the most widely used and effective defence, especially for FGSM-style perturbations. However, it tends to overfit to known attacks and requires substantial computational resources for training.
- *Defensive distillation* offers moderate robustness by smoothing decision boundaries, yet it is easily circumvented by stronger attacks (e.g., C&W), as it inadvertently introduces gradient obfuscation.
- *Gradient masking*, though computationally inexpensive, often leads to poor generalisability and has been demonstrated to be ineffective under adaptive threat models.
- *Randomised smoothing* provides theoretical robustness bounds under certain perturbation norms, but incurs substantial latency at inference and has limited practical adoption.
- *Ensemble adversarial training* improves transfer robustness and mitigates overfitting to a specific attack strategy, albeit at increased training cost.

Effectiveness of individual defence strategies depends on the threat model, available computational budget, and tolerance for inference latency. The diversity of trade-offs requires defence-in-depth architectures that combine multiple strategies for layered robustness.

## 17 Discussion

Empirical evidence accumulated over the past decade shows that comprehensive knowledge of a model’s internals is no longer a prerequisite for producing high-confidence adversarial failures. Gradient-free bandit optimisers, finite-difference estimators, and transfer-based strategies now attain misclassification rates on ImageNet that approach those of canonical white-box attacks, demonstrating that decision boundaries learned by modern architectures remain highly correlated even when the parameters are hidden. This observation calls into question evaluation protocols that rely exclusively on fast, single-step perturbations such as FGSM or on a limited set of norm-bounded iterative attacks.

Robustness is also demonstrably task-dependent. Adversarial training with projected-gradient descent improves *ε* = 8/255 accuracy on CIFAR-10 to roughly 47%, yet under identical perturbation budgets the same procedure affords fewer than 20% robust accuracy on ImageNet. When the perturbations are extended from pixel noise to geometric distortions, two-degree rotations or sub-pixel translations in autonomous-driving imagery, the effective accuracy drop is larger still, pointing to a gap between standard benchmark metrics and the failure modes that dominate physical deployments.

Privacy leakage scales with both model capacity and the granularity of the outputs released to the user. Membership-inference and model-inversion attacks achieve 60–70% accuracy on unprotected CIFAR-10 classifiers but fall to near-random levels once training is performed with differential-privacy stochastic gradient descent at ε ≈ 1. The modest two-to-four-percentage-point reduction in clean accuracy observed in these experiments suggests that rigorous privacy guarantees and practical utility are not mutually exclusive.

No single defensive mechanism exhibits universal efficacy. Defensive distillation, feature squeezing, and gradient masking all deteriorate under adaptive evaluation; only multi-layered strategies that combine adversarial training, randomised smoothing, differential privacy, and interface hardening provide measurable resilience, and even these solutions leave a gap of more than 50 percentage points between clean and robust accuracy on large-scale image classification. Large-language models present an additional alignment problem: automated red-teaming frameworks such as MAD-MAX attain jailbreak success rates exceeding 95 % on GPT-4-class systems with an average of 11 queries, revealing that reinforcement-learning-based alignment alone is insufficient to prevent malicious prompt injection.

Finally, supply-chain attacks on pre-trained weights, data-set poisoning in self-supervised learning, and logic bombs embedded in parameter-efficient adapters highlight that adversarial robustness must be considered across the entire model lifecycle, data acquisition, training, distribution, and inference monitoring, rather than at a single frozen checkpoint.

## 18 Conclusion

This work advances the study of adversarial AI security by reframing robustness as a system-level property of agentic artificial intelligence rather than a narrowly defined characteristic of individual models or inputs. By formalising adversarial risk across perceptual, cognitive, and executive layers, the proposed framework extends classical adversarial machine-learning theory to account for autonomy, self-governance, and closed-loop decision-making. This shift is essential for analysing modern AI systems whose behaviour emerges over time through feedback, planning, and interaction with dynamic environments.

A central insight of this study is that adversarial vulnerabilities cannot be fully understood, or mitigated, through input-level defences alone. While perceptual attacks remain the most empirically validated, higher-order failures arise when erroneous perceptions propagate through internal reasoning, policy formation, and actuation. The analysis demonstrates that architectural properties such as feedback dynamics, memory, and goal specification fundamentally shape adversarial behaviour, transferability, and impact. Consequently, robustness must be treated as an emergent property of the entire agent–environment system rather than as a static performance metric.

Importantly, this paper does not position higher-order agentic adversarial attacks as empirically settled phenomena. Instead, they are framed as hypothesis-driven, architecturally motivated risks that demand systematic investigation. The absence of standardised benchmarks for autonomy, long-horizon decision-making, and behavioural integrity represents a critical gap in current research. Addressing this gap requires new evaluation environments, metrics that capture cumulative behavioural deviation and policy drift, and defence mechanisms that integrate perceptual robustness with governance, verification, and runtime oversight.

Beyond consolidating existing adversarial knowledge, the primary contribution of this work lies in defining a coherent research agenda for agentic AI security. By unifying adversarial machine learning, systems safety, and control-theoretic perspectives, the framework provides a foundation for studying resilience, stability, and alignment in autonomous AI systems. As AI agents are increasingly deployed in safety-critical and socially consequential contexts, such system-level approaches will be indispensable for ensuring not only accuracy, but trustworthy and accountable behaviour over time.

## Data Availability

The original contributions presented in the study are included in the article/supplementary material, further inquiries can be directed to the corresponding author.

## Footnotes

1
robust-ml.org

2
https://www.tensorflow.org/versions/r2.0/api_docs/python/tf/keras/applications/MobileNetV2

3
https://image-net.org/
